# Supplementary material for: Genome-wide detection of cytosine methylations in plant from Nanopore data using deep learning
Source: Nat Commun. 2021 Oct 13;12:5976. doi: 10.1038/s41467-021-26278-9 (PMC8514461; doi:10.1038/s41467-021-26278-9)
Supplement: Supplementary file 1 — Supplementary Information [file 41467_2021_26278_MOESM1_ESM.pdf]

# Genome-wide detection of cytosine methylations in plant from Nanopore data using deep learning

Ni *et al.*

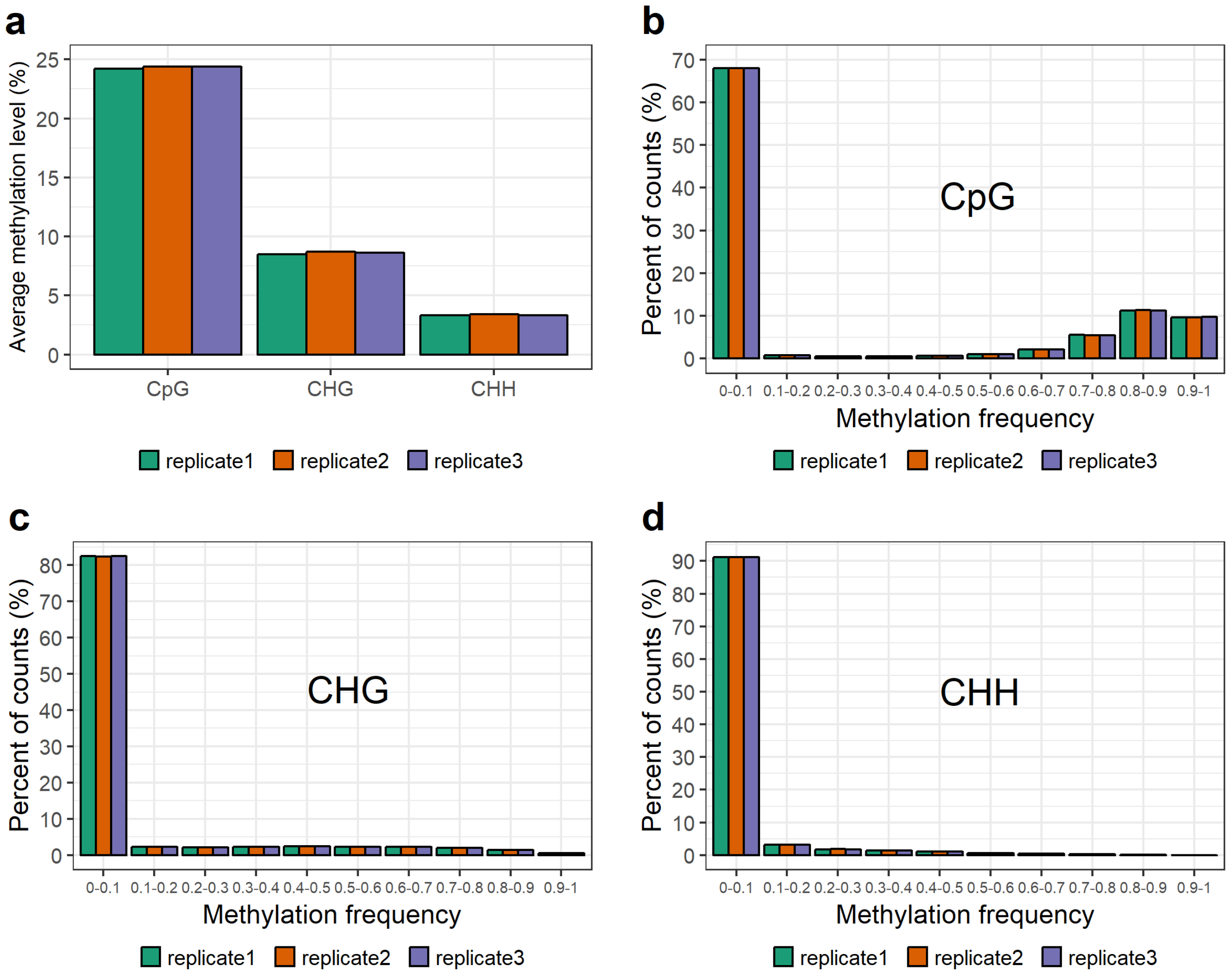


**Supplementary Fig. 1.** General pattern of 5mC methylation in *A. thaliana* from bisulfite sequencing (3 technical replicates: replicate1, replicate2, and replicate3). **a:** Genome average levels of 5mC (CpG, CHG, CHH) methylation in *A. thaliana*. **b-d**: Distribution of methylation frequency of CpG (b), CHG (c), and CHH (d). The x-axis is divided into 10 bins. The y-axis is the percent of total counts for each bin respectively. When calculating methylation frequency, only the sites with at least 5 mapped reads are considered.

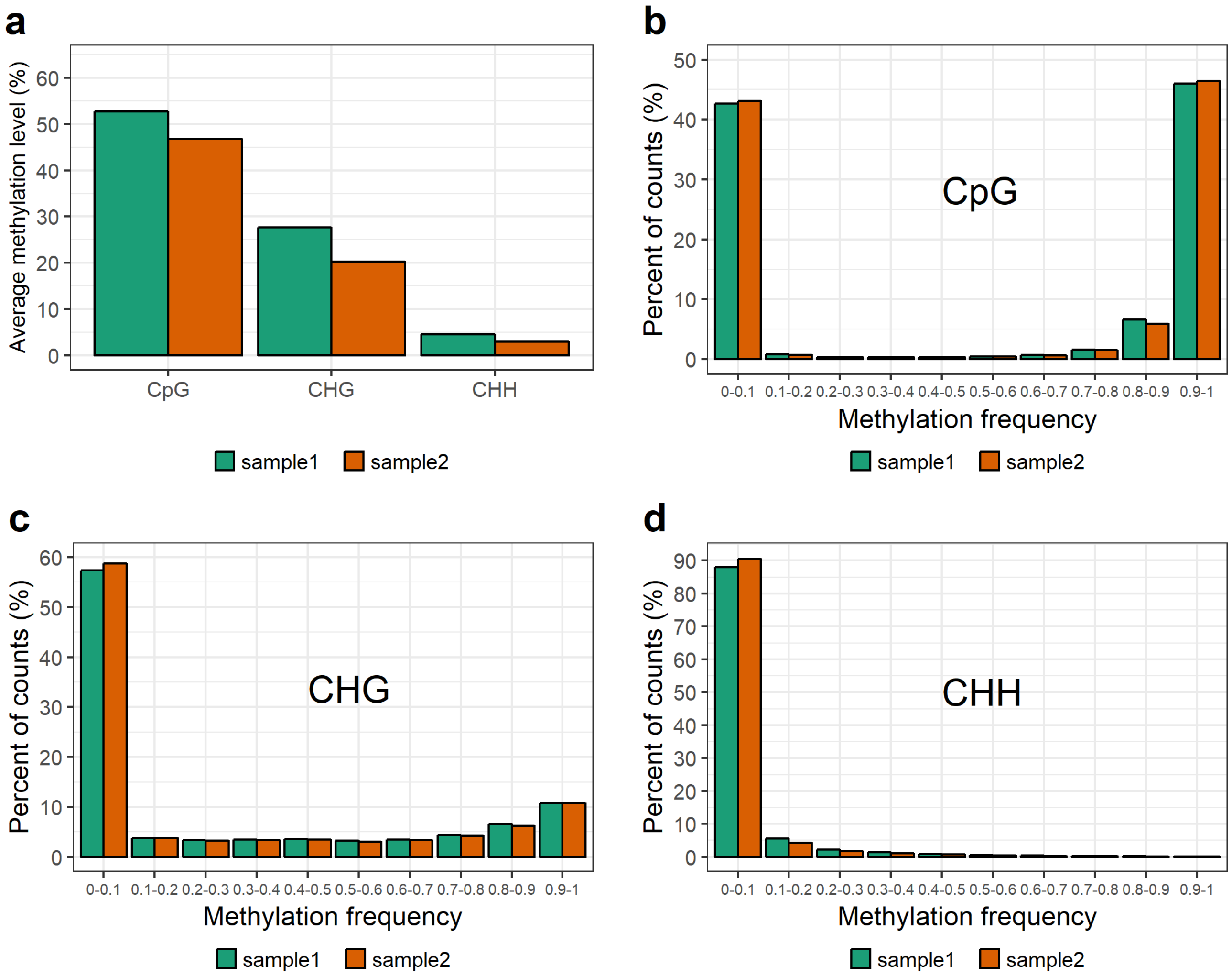


**Supplementary Fig. 2.** General pattern of 5mC methylation in *O. sativa* from bisulfite sequencing (2 biological replicates: sample1 and sample2). **a:** Genome average levels of 5mC (CpG, CHG, CHH) methylation in *O. Sativa*. **b-d**: Distribution of methylation frequency of CpG (b), CHG (c), and CHH (d). The x-axis is divided into 10 bins. The y-axis is the percent of total counts for each bin respectively. When calculating methylation frequency, only the sites with at least 5 mapped reads are considered.

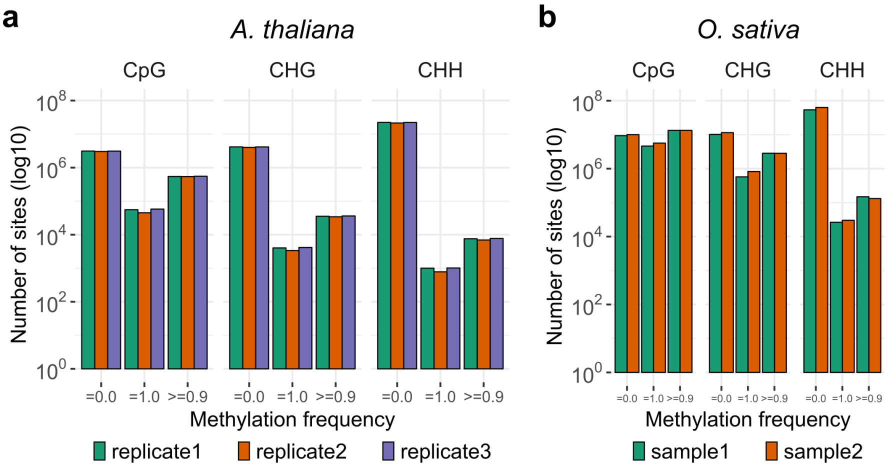


**Supplementary Fig. 3.** Number of fully unmethylated sites, fully methylated sites, sites of which methylation frequency>=0.9 based on bisulfite sequencing. **a:** *A. thaliana*. **b:** *O. sativa*.

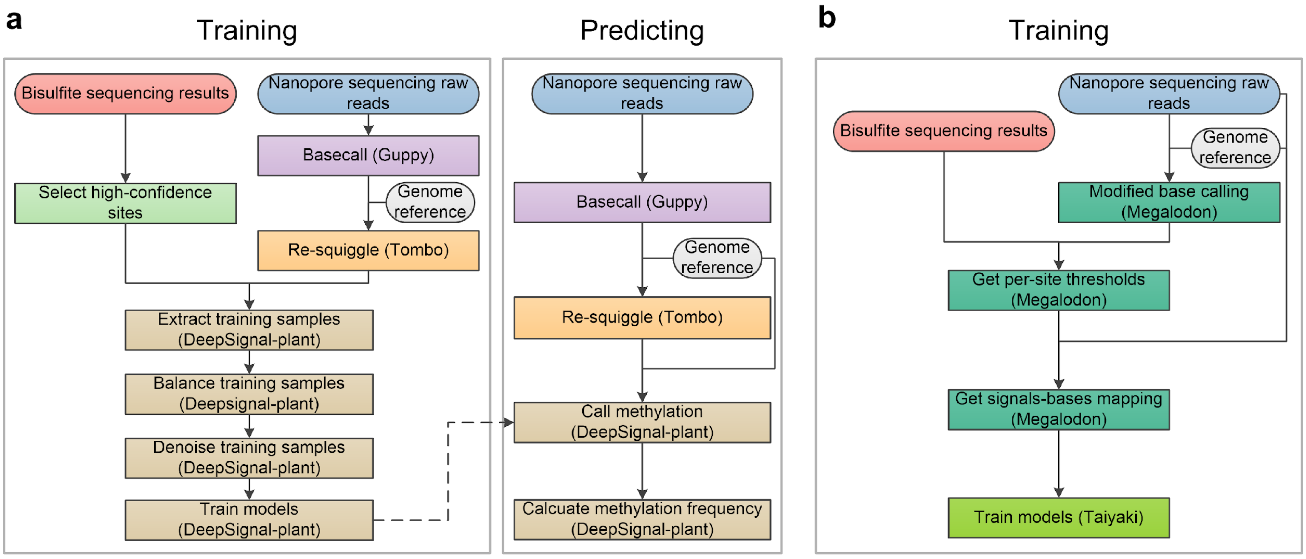


**Supplementary Fig. 4.** Flowchart of our proposed pipeline (DeepSignal-plant) and Megalodon. **a:** Training and predicting process of DeepSignal-plant. **b:** Training process of Megalodon.

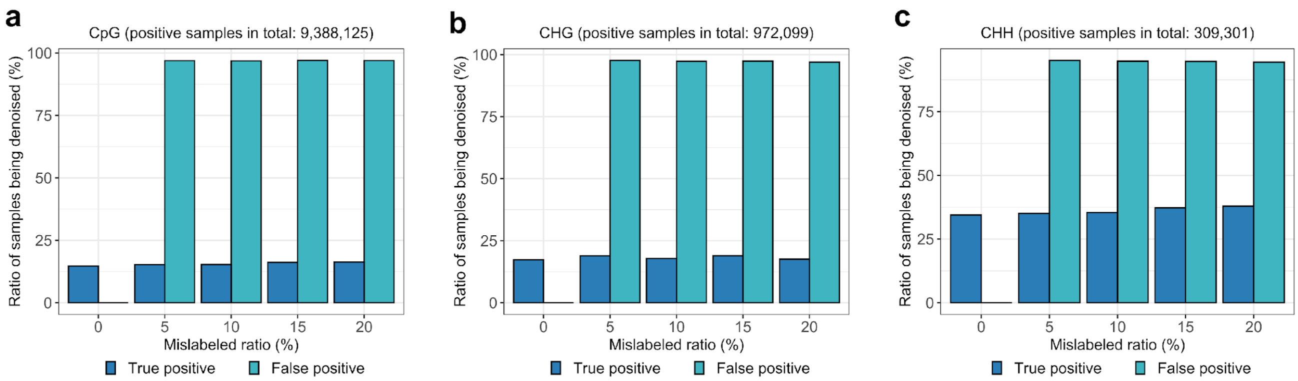


**Supplementary Fig. 5.** Simulation experiment of the denoising method. **a-c:** Ratio of positive samples denoised by the denoising method for CpG (a), CHG (b), and CHH (c). Values in the plots are averages of 5 repeated tests.

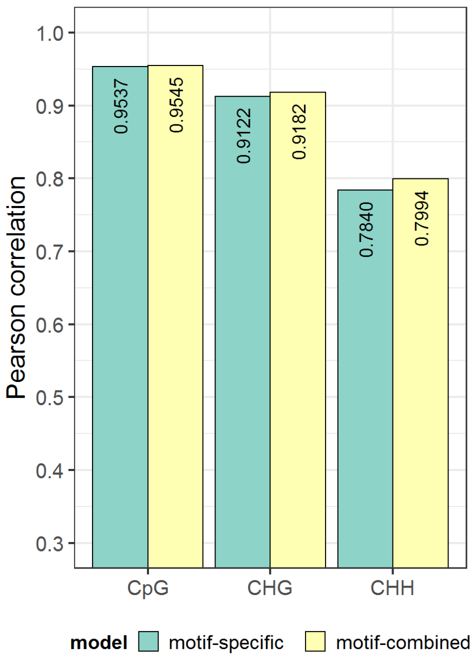


**Supplementary Fig. 6.** Comparison of motif-specific models and the motif-combined model of DeepSignal-plant on 20× *A. thaliana* reads.

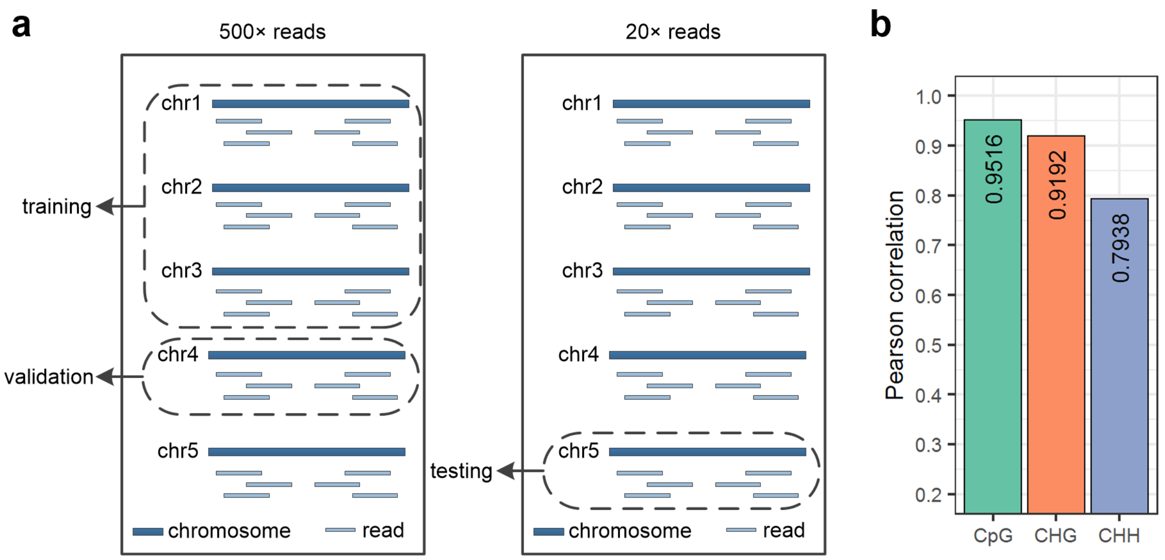


**Supplementary Fig. 7.** Chromosomal cross-validation of DeepSignal-plant using *A. thaliana* data. **a:** Data partition for the cross-validation. **b:** Performance of DeepSignal-plant in the cross-validation.

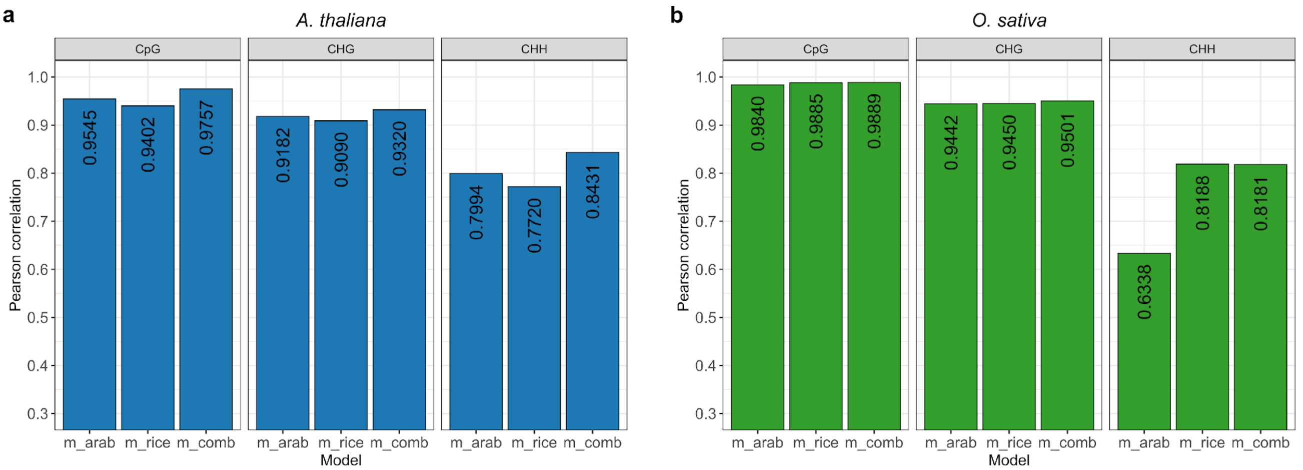


**Supplementary Fig. 8.** 5mC detection in *A. thaliana* (**a**) and *O. sativa* (**b**) using models of DeepSignal-plant trained from different datasets. m_arab, m_rice, m_comb represent the models of DeepSignal-plant trained using ~500× *A. thaliana* Nanopore reads, ~115× *O. sativa* (sample1) Nanopore reads and the combined Nanopore reads, respectively. Pearson correlations are calculated using the results from ~20× Nanopore reads of *A.thaliana* and *O. sativa* (sample1) with the corresponding bisulfite replicates, respectively.

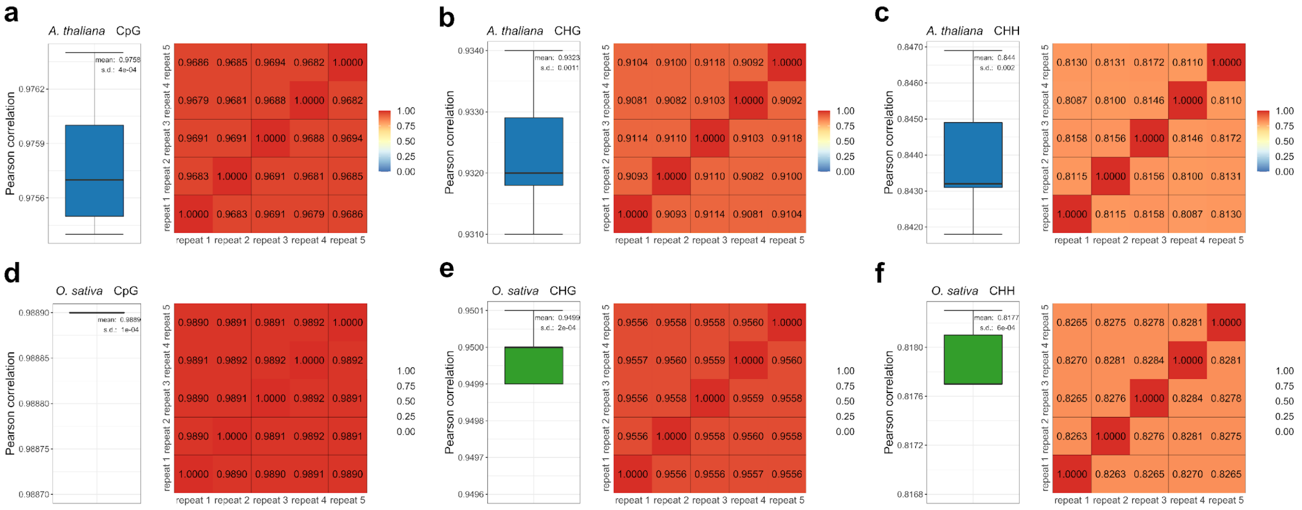


**Supplementary Fig. 9.** Evaluation of our proposed pipeline by randomly selecting ~20× reads of *A. thaliana* and *O. sativa* (sample1) for 5 repeated times. **a-c:** CpG (a), CHG (b), and CHH (c) methylation of *A. thaliana*. **d-f:** CpG (a), CHG (b), and CHH (c) methylation of *O. sativa* (sample1). Boxplot: Pearson correlation with the results of bisulfite sequencing. $n$ = 5 repeated experiments; Boxplots indicate 50th percentile (middle line), 25th and 75th percentile (box), the smallest value within 1.5 times the interquartile range below 25th percentile, and the largest value within 1.5 times the interquartile range above 75th percentile (whiskers). Heatmap: Pearson correlation between the results of the 5 repeated tests. Models of DeepSignal-plant were trained using combined reads of *A. thaliana* and *O. sativa*.

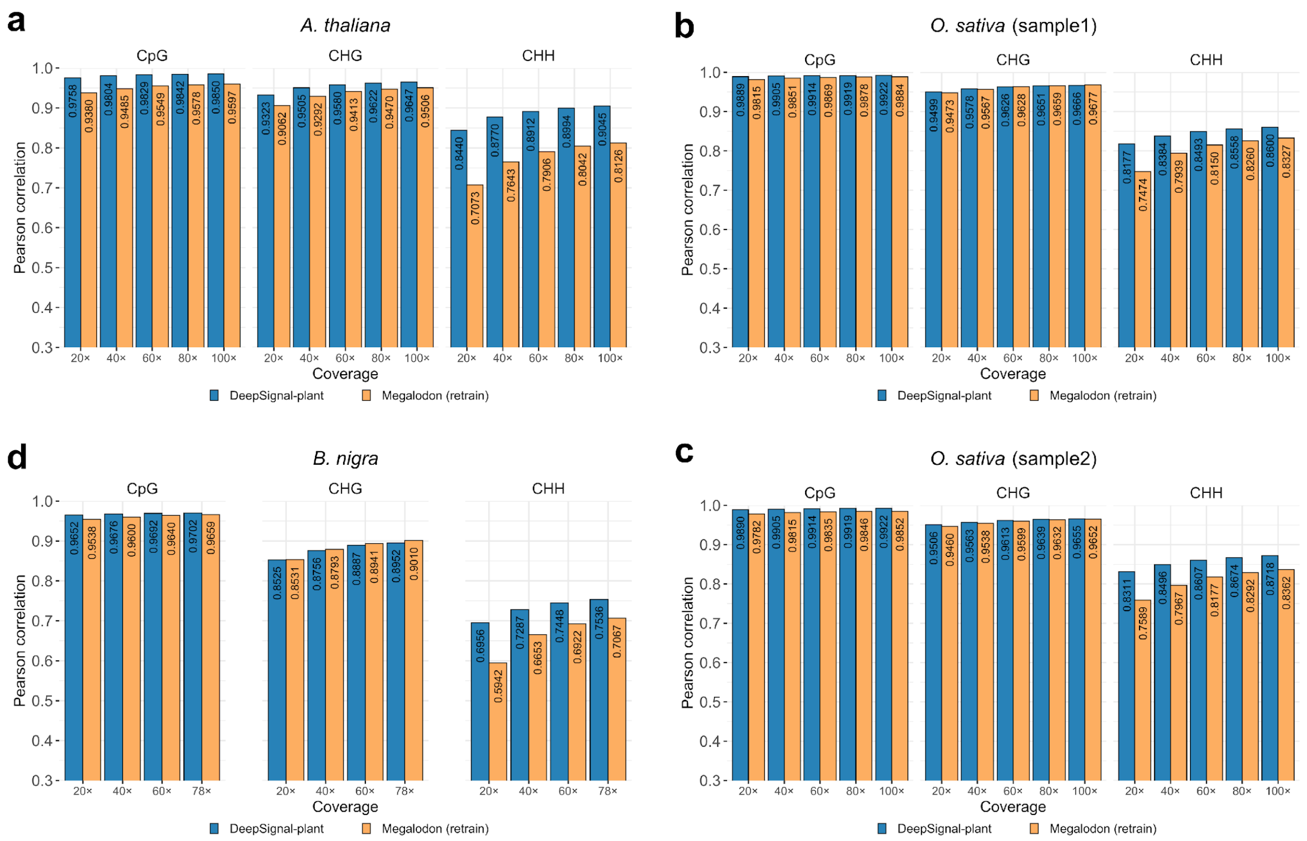


**Supplementary Fig. 10.** Comparison between DeepSignal-plant and Megalodon against bisulfite sequencing on 5mC detection under different coverages of Nanopore reads in *A. thaliana* (**a**), *O. sativa* (sample1) (**b**), *O. sativa* (sample2) (**c**), and *B. nigra* (**d**). For each coverage (20× to 80× for *A. thaliana* and *O. sativa*, 20× to 60× for *B. nigra*), the reads were randomly shuffled and selected from the whole ~100×/78× reads. Values for 20×, 40×, 60×, and 80× are averages of 5 replicated tests. Models of Megalodon and DeepSignal-plant were trained using combined reads of *A. thaliana* and *O. sativa*.

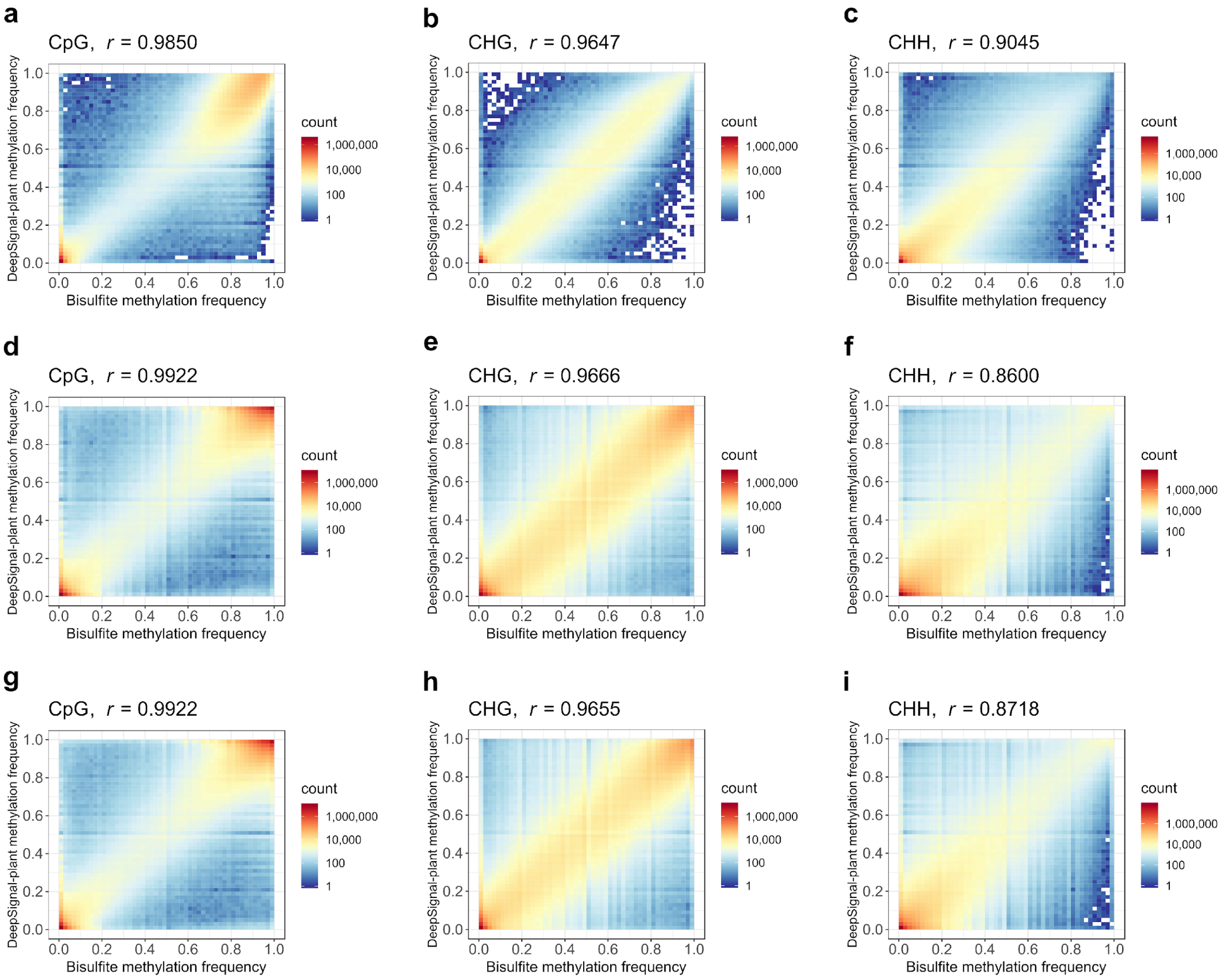


**Supplementary Fig. 11.** Comparison of methylation frequencies of cytosines calculated by DeepSignal-plant and bisulfite sequencing. **a-c:** CpG (a), CHG (b), and CHH (c) methylation in *A. thaliana*. **d-f:** CpG (d), CHG (e), and CHH (f) methylation in *O. sativa* (sample1). **g-i:** CpG (g), CHG (h), and CHH (i) methylation in *O. sativa* (sample2). *r* is Pearson correlation. ~100× coverage of Nanopore reads was used.

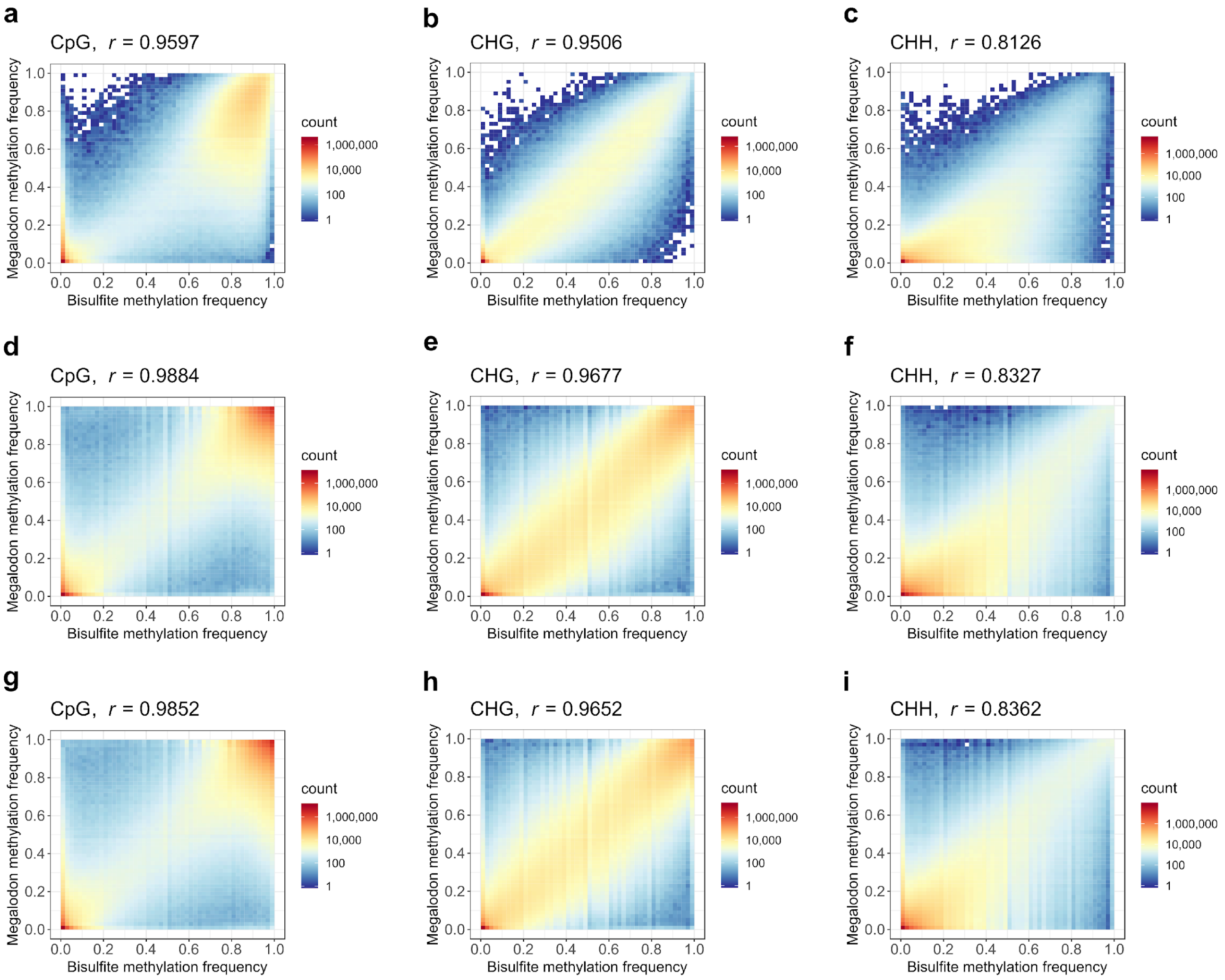


**Supplementary Fig. 12.** Comparison of methylation frequencies of cytosines calculated by Megalodon and bisulfite sequencing. **a-c:** CpG (a), CHG (b), and CHH (c) methylation in *A. thaliana*. **d-f:** CpG (d), CHG (e), and CHH (f) methylation in *O. sativa* (sample1). **g-i:** CpG (g), CHG (h), and CHH (i) methylation in *O. sativa* (sample2). *r* is Pearson correlation. ~100× coverage of Nanopore reads was used. Models of Megalodon were trained using combined reads of *A. thaliana* and *O. sativa*.

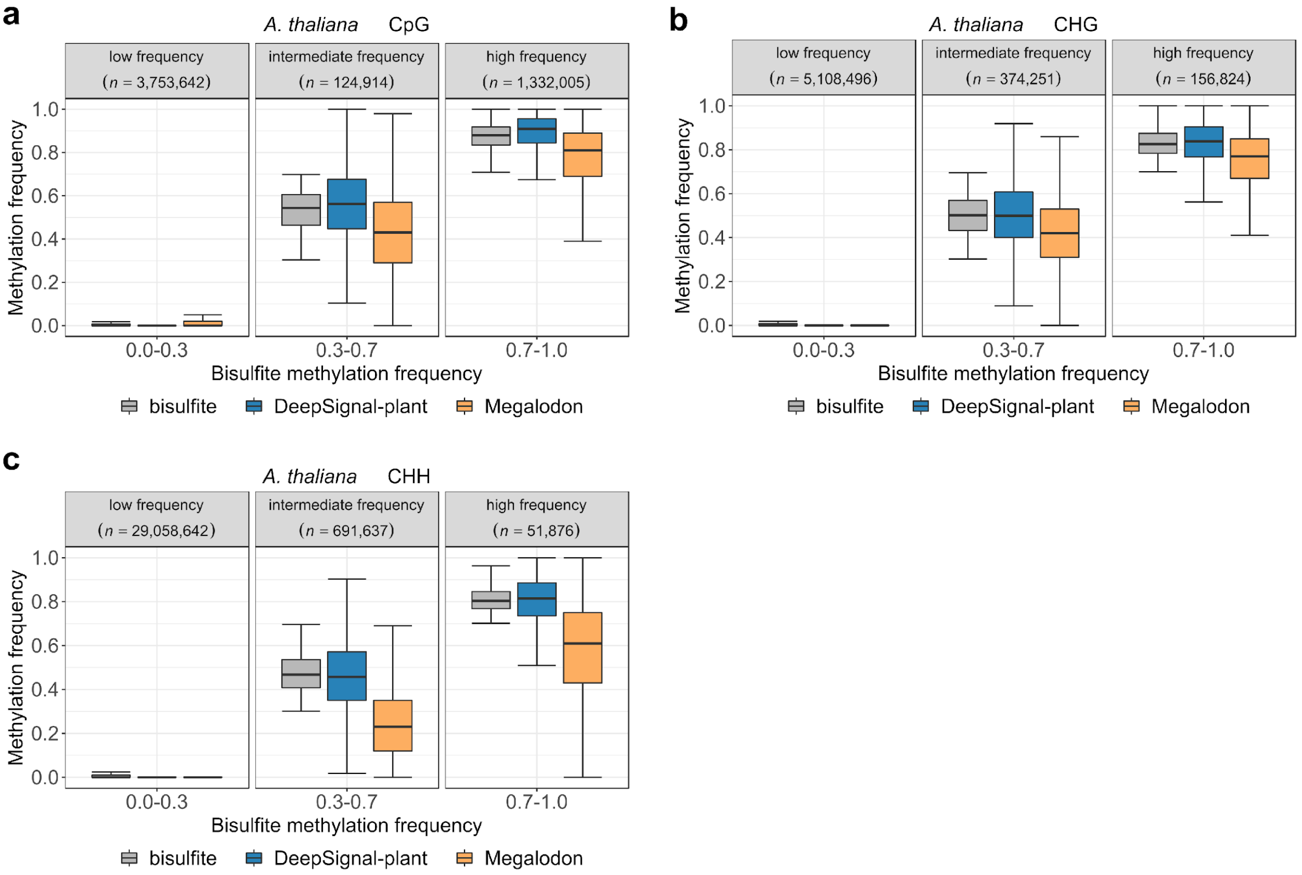


**Supplementary Fig. 13.** Distribution of methylation frequencies called by DeepSignal-plant and Megalodon from ~100× Nanopore reads of *A. thaliana* against bisulfite sequencing across three methylation bins: low frequency (0.0-0.3), intermediate frequency (0.3-0.7), and high frequency (0.7-1.0). **a-c**: CpG (a), CHG (b), and CHH (c) methylation. Models of Megalodon and DeepSignal-plant were trained using combined reads of *A. thaliana* and *O. sativa*. *n* = number of cytosines in each methylation bin; Boxplots indicate 50th percentile (middle line), 25th and 75th percentile (box), the smallest value within 1.5 times the interquartile range below 25th percentile, and largest value within 1.5 times the interquartile range above 75th percentile (whiskers).

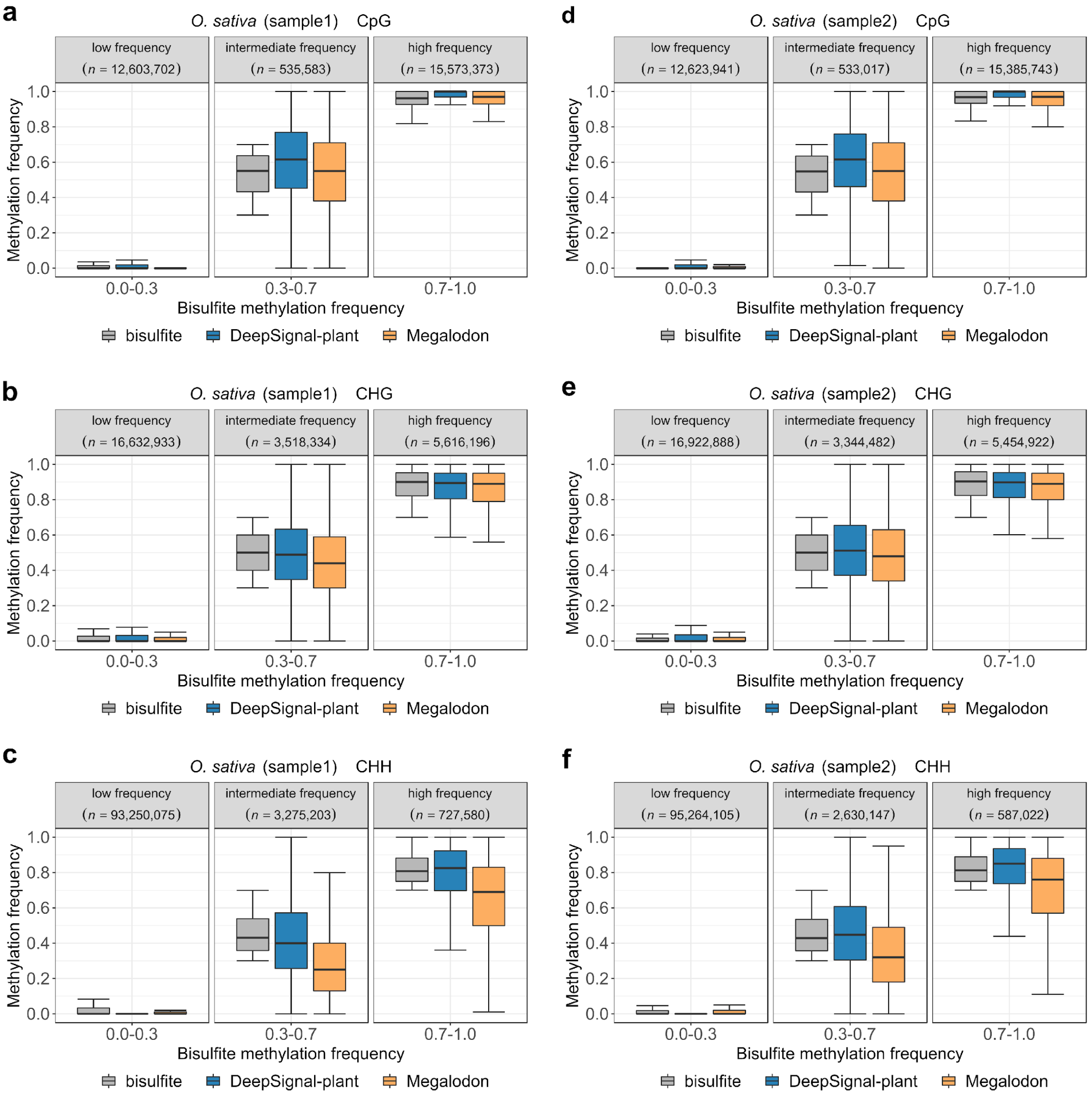


**Supplementary Fig. 14.** Distribution of methylation frequencies called by DeepSignal-plant and Megalodon from ~100× Nanopore reads of *O. sativa* (sample1) and *O. sativa* (sample2), respectively, against bisulfite sequencing across three methylation bins: low frequency (0.0-0.3), intermediate frequency (0.3-0.7), and high frequency (0.7-1.0). **a-c**: CpG (a), CHG (b), and CHH (c) methylation in *O. sativa* (sample1). **d-f**: CpG (d), CHG (e), and CHH (f) methylation in *O. sativa* (sample2). Models of Megalodon and DeepSignal-plant were trained using combined reads of *A. thaliana* and *O. sativa*. *n* = number of cytosines in each methylation bin; Boxplots indicate 50th percentile (middle line), 25th and 75th percentile (box), the smallest value within 1.5 times the interquartile range below 25th percentile and largest value within 1.5 times interquartile range above 75th percentile (whiskers).

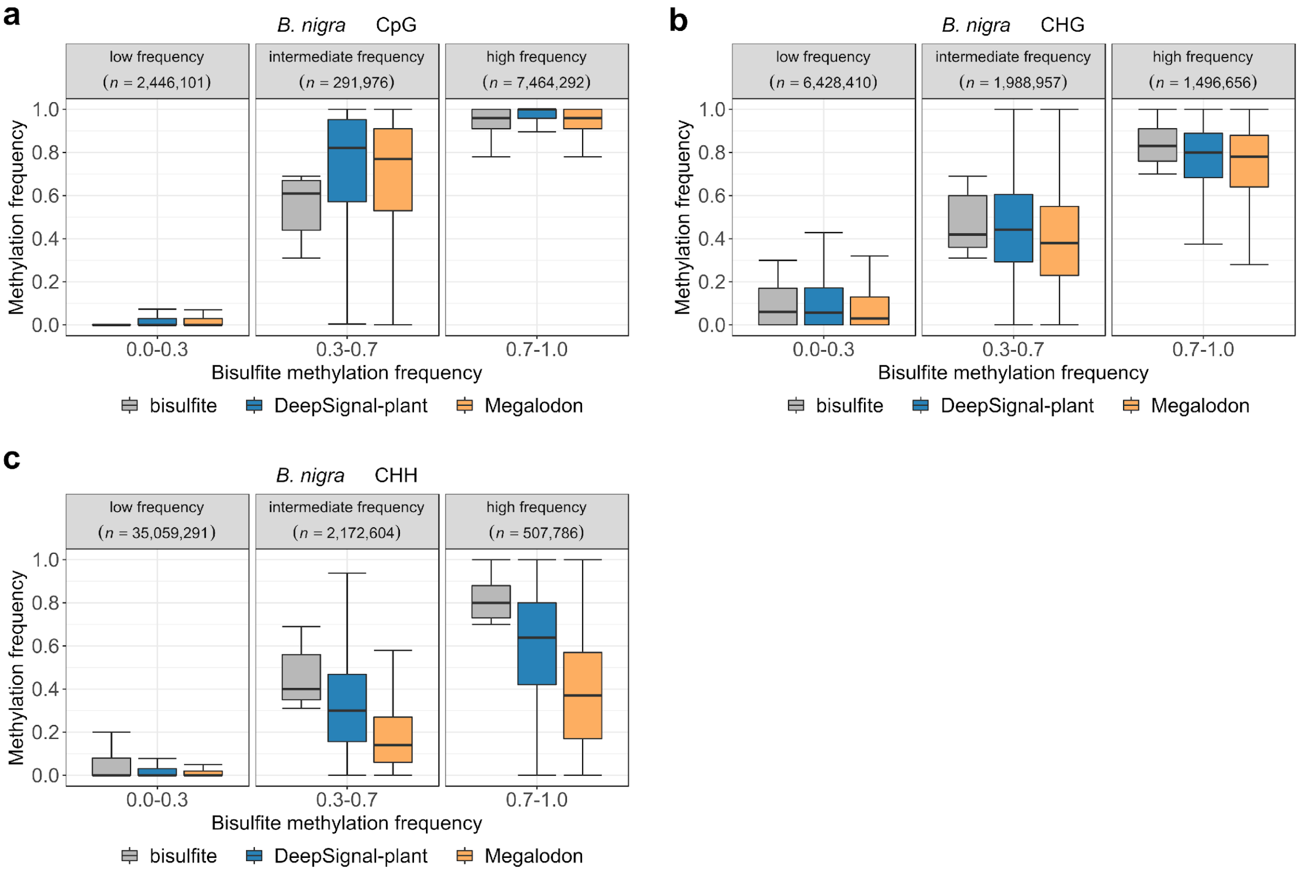


**Supplementary Fig. 15.** Distribution of methylation frequencies predicted by DeepSignal-plant and Megalodon from ~78× Nanopore reads of *B. nigra* against bisulfite sequencing across three methylation bins: low frequency (0.0-0.3), intermediate frequency (0.3-0.7), and high frequency (0.7-1.0). **a**: CpG motif. **b**: CHG motif. **c**: CHH motif. Models of DeepSignal-plant and Megalodon were trained using combined reads of *A. thaliana* and *O. sativa*. *n* = number of cytosines in each methylation bin; Boxplots indicate 50th percentile (middle line), 25th and 75th percentile (box), the smallest value within 1.5 times the interquartile range below 25th percentile, and largest value within 1.5 times the interquartile range above 75th percentile (whiskers).

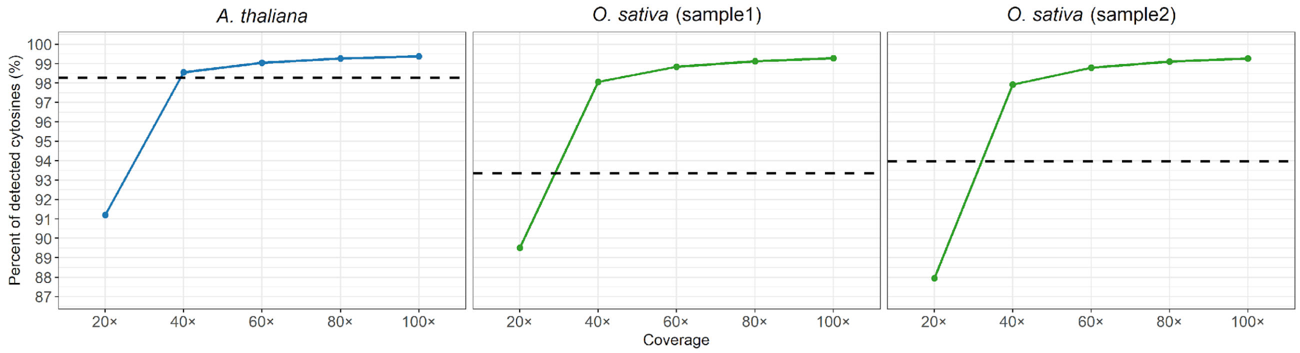


**Supplementary Fig. 16.** Percent of cytosines detected by DeepSignal-plant from Nanopore sequencing (coverage>=5) in genomes of *A. thaliana* and *O. sativa*. Reads for coverage 20× to 80× are randomly shuffled and selected from ~100× reads. Values for coverage 20× to 80× are averages of 5 replicated tests. Black dash lines indicate the percent of cytosines detected by bisulfite sequencing (coverage>=5).

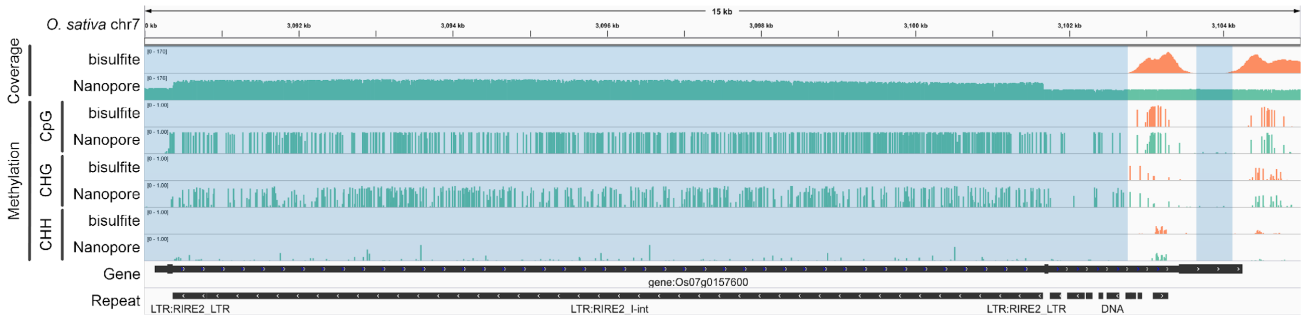


**Supplementary Fig. 17.** Genome browser view of the reads coverage and methylation in a 15 kb region (chr7:3089990-3104990:+) of *O. sativa* (sample2) detected by bisulfite sequencing (Bismark) and Nanopore sequencing (DeepSignal-plant). The blue shaded area shows the gaps which cannot be mapped by bisulfite sequencing. Source data are provided as a Source Data file.

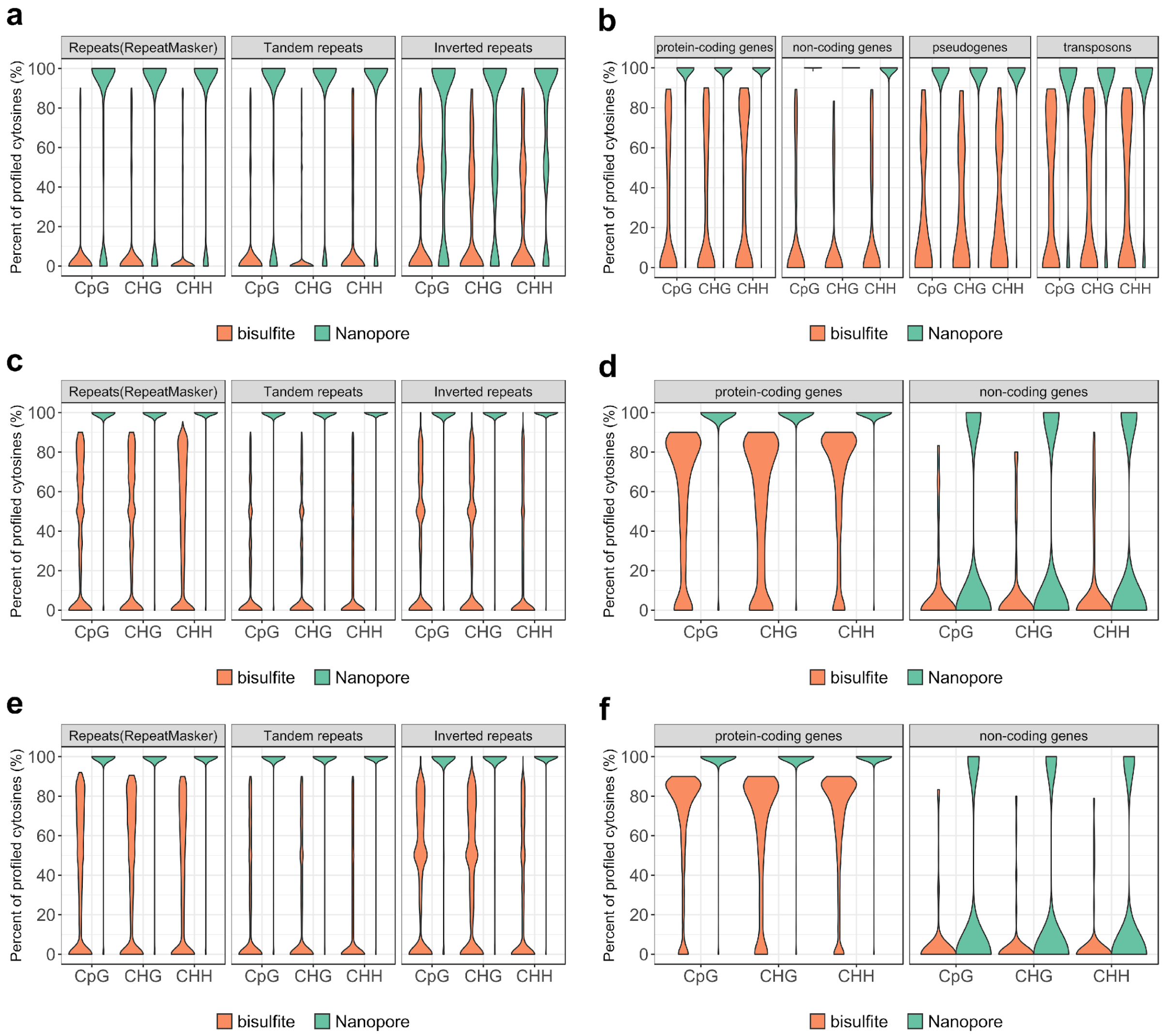


**Supplementary Fig. 18.** Percent of profiled cytosines by bisulfite sequencing (Bismark) and Nanopore sequencing (DeepSignal-plant) in biological regions that cannot be fully profiled by bisulfite sequencing. Repeat regions and gene regions in which the percent of profiled cytosines by bisulfite sequencing <= 90% are selected for comparison. **a-b:** Comparison of repeat regions (a) and gene regions (b) in *A. thaliana*. **c-d:** Comparison of repeat regions (c) and gene regions (d) in *O. sativa* (sample1). **e-f:** Comparison of repeat regions (e) and gene regions (f) in *O. sativa* (sample2).

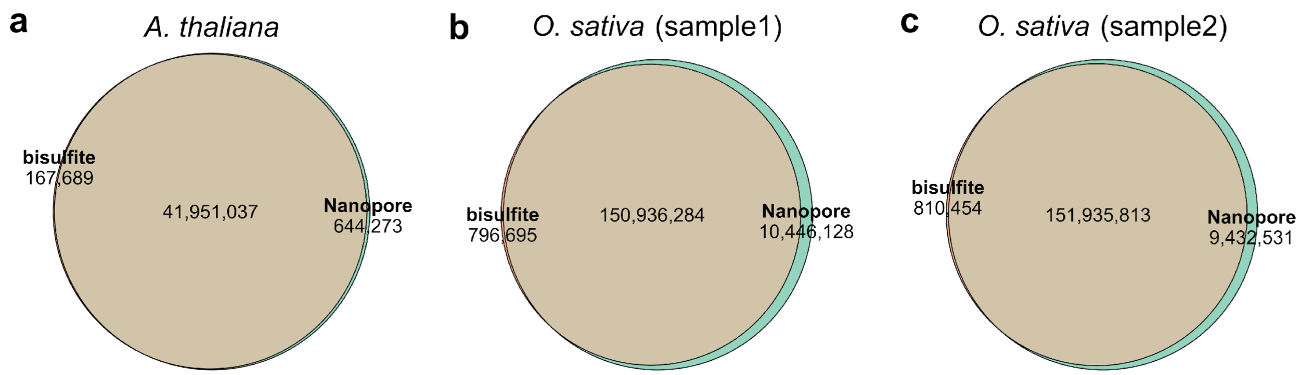


**Supplementary Fig. 19.** Comparison of cytosines detected by bisulfite sequencing (Bismark) and Nanopore sequencing (DeepSignal-plant, ~100×). **a:** A. thaliana. **b:** *O. sativa* (sample1). **c:** *O. sativa* (sample2)

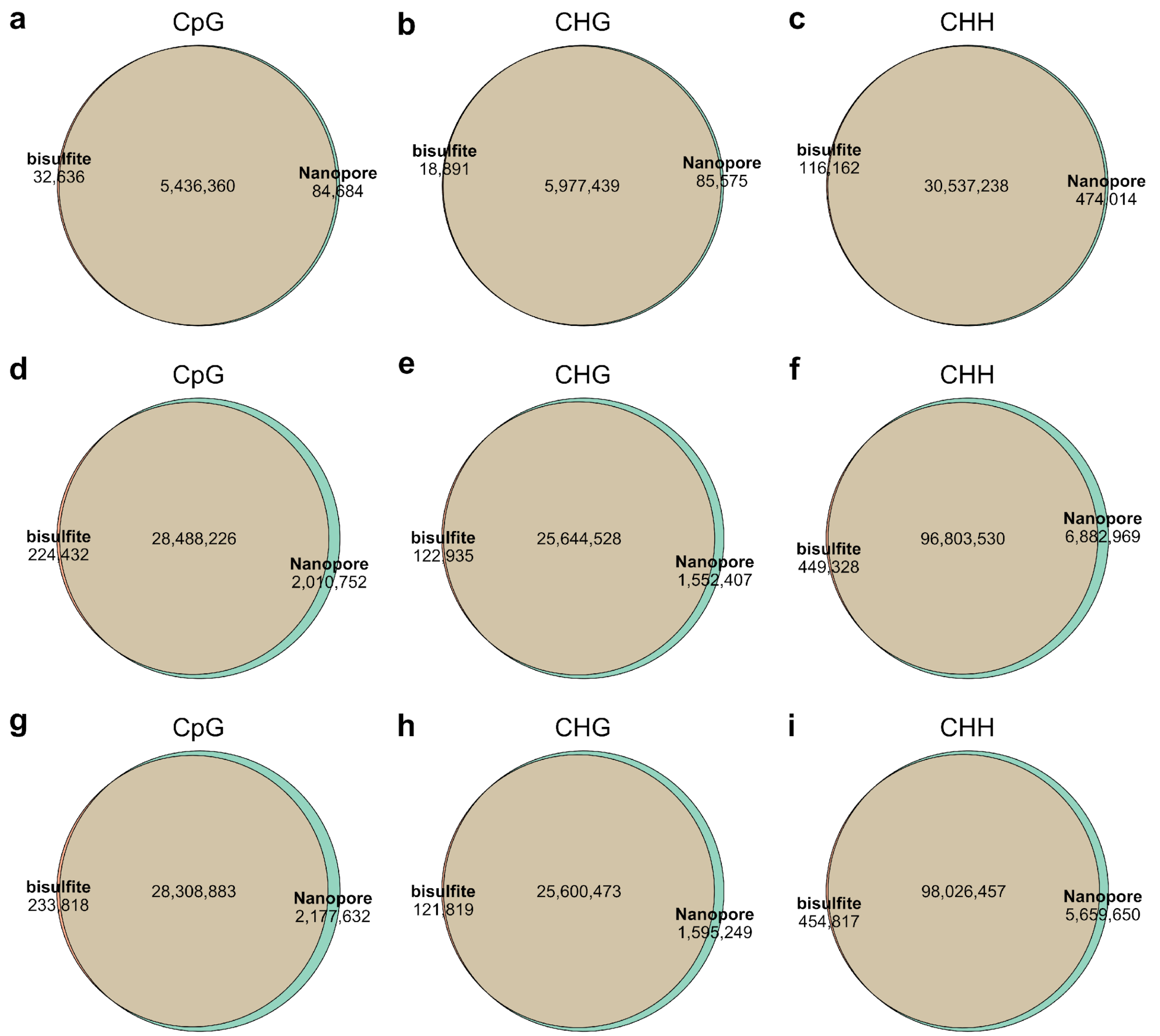


**Supplementary Fig. 20.** Comparison of cytosines detected by bisulfite sequencing (Bismark) and Nanopore sequencing (DeepSignal-plant, ~100×) in three motifs. **a-c**: Comparison of the number of CpG (a), CHG (b), and CHH (c) sites in *A. thaliana*. **d-f:** Comparison of the number of CpG (d), CHG (e), and CHH (f) sites in *O. sativa* (sample1). **g-i:** Comparison of the number of CpG (g), CHG (h), and CHH (i) sites in *O. sativa* (sample2).

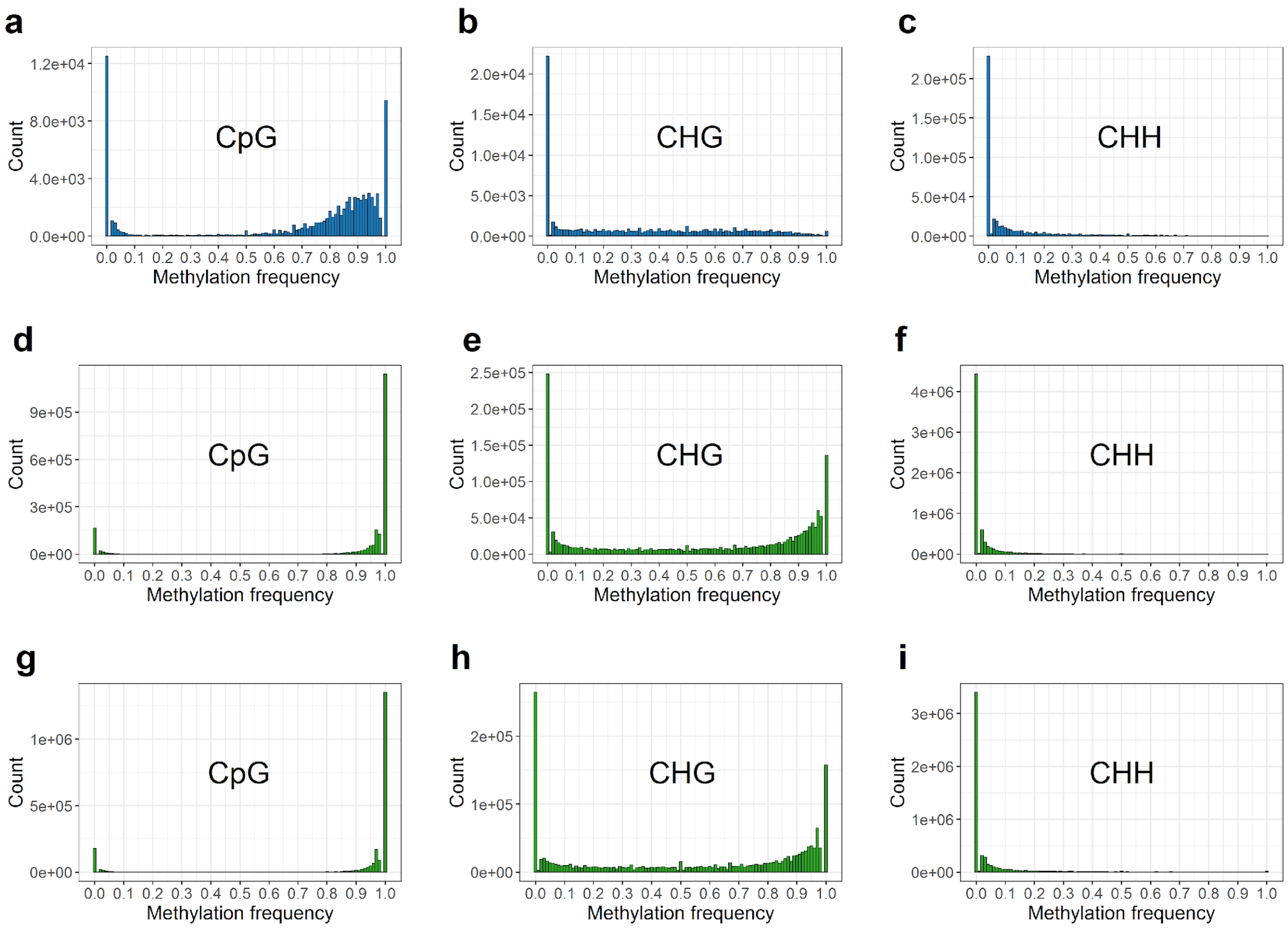


**Supplementary Fig. 21.** Methylation frequencies of cytosines which can only be detected by Nanopore sequencing (DeepSignal-plant). **a-c:** Methylation frequencies of CpG (a), CHG (b), and CHH (c) sites in *A. thaliana*. **d-f:** Methylation frequencies of CpG (d), CHG (e), and CHH (f) sites in *O. sativa* (sample1). **g-i:** Methylation frequencies of CpG (g), CHG (h), and CHH (i) sites in *O. sativa* (sample2).

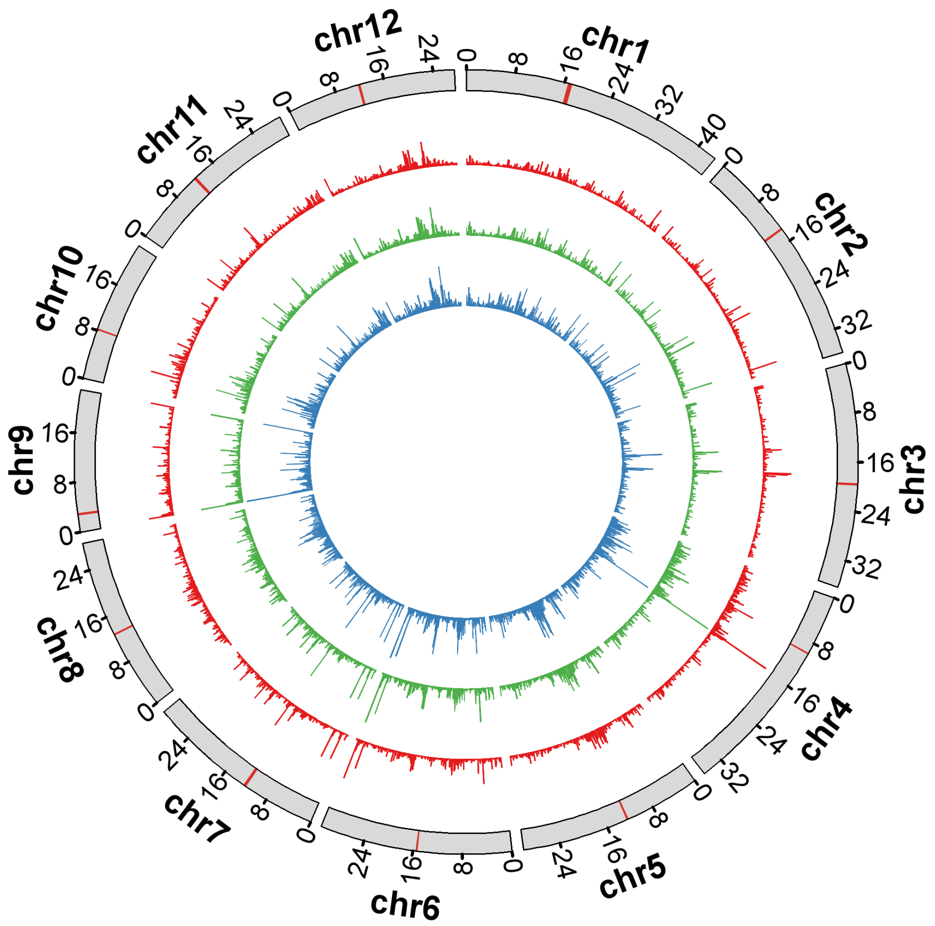


**Supplementary Fig. 22.** Circos plot of the number of cytosines detected by Nanopore sequencing only in the *O. sativa* (sample2). Cycles from inner to outer: CpG (blue), CHG (green), CHH (red), reference (the chromosomes are binned into 200,000-bp (base pair) windows. The centromeric region is indicted by the red bar in each chromosome). Source data are provided as a Source Data file.

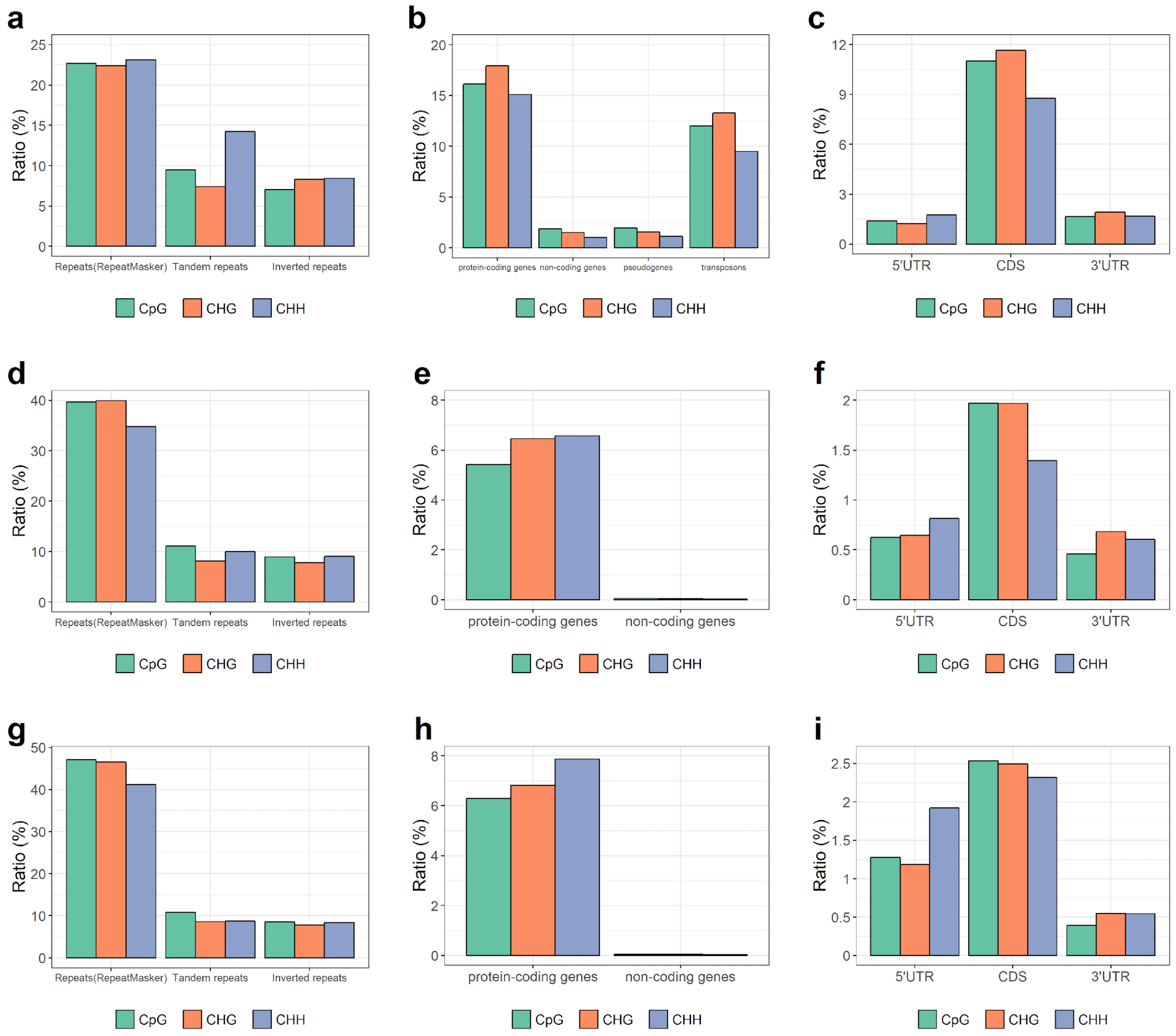


**Supplementary Fig. 23.** Distribution of cytosines which can only be detected by Nanopore sequencing (DeepSignal-plant) in repeats and gene regions. **a-c:** Proportion of cytosines which can only be detected by Nanopore sequencing in repeat regions (a), different kinds of genes (b), and gene bodies (c) of *A. thaliana*. **d-f:** Proportion of cytosines which can only be detected by Nanopore sequencing in repeat regions (d), different kinds of genes (e), and gene bodies (f) of *O. sativa* (sample1). **g-i:** Proportion of cytosines which can only be detected by Nanopore sequencing in repeat regions (g), different kinds of genes (h), and gene bodies (i) of *O. sativa* (sample2).

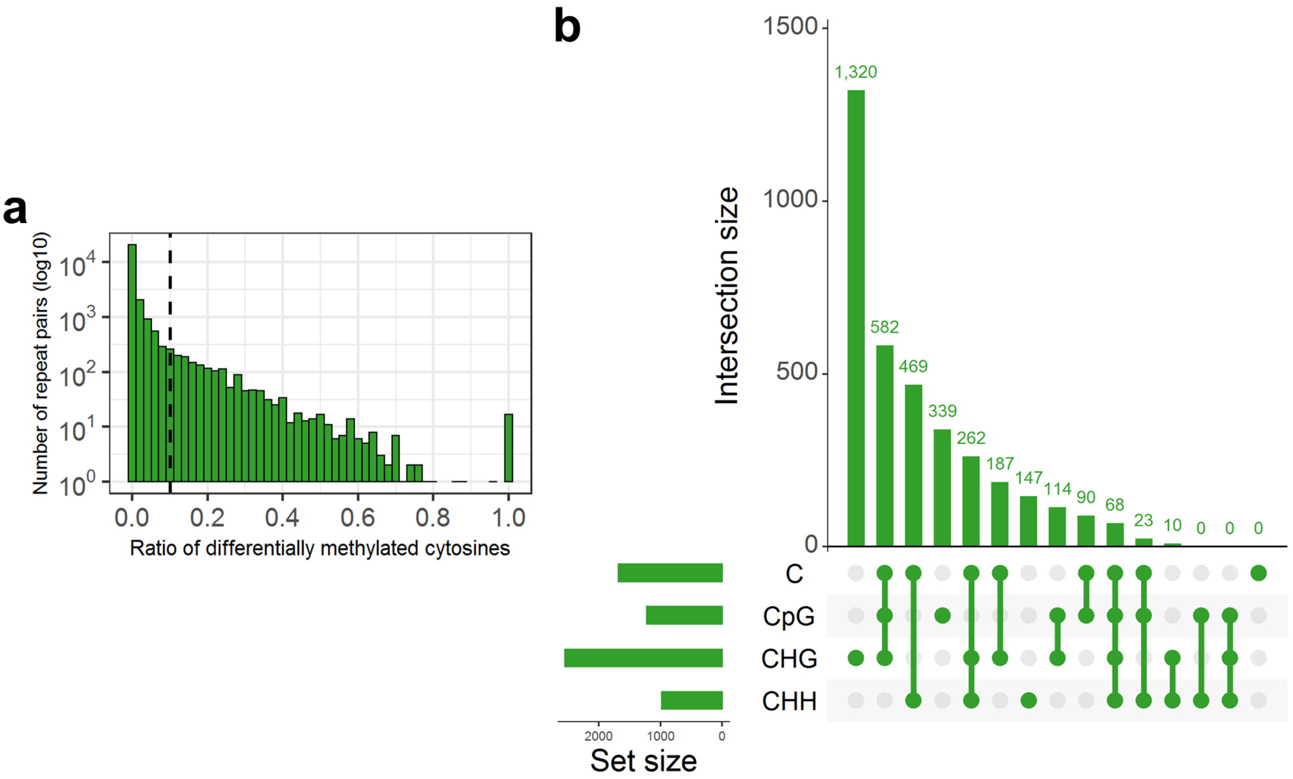


**Supplementary Fig. 24.** Our proposed pipeline identified differentially methylated repeat pairs in *O. sativa* (sample2). **a:** Ratio of differentially methylated cytosines to total cytosines in each repeat pair. The black dash lines (10%) indicate repeat pairs are differentially methylated (right) or not (left). **b:** Matrix layout for all intersections of four sets of differentially methylated repeat pairs profiled by cytosines, CpG sites, CHG sites, and CHH sites independently. Circles in the matrix indicate sets that are part of the intersection; the up bars indicate the size of each intersection; the left bars indicate the total size of each set.

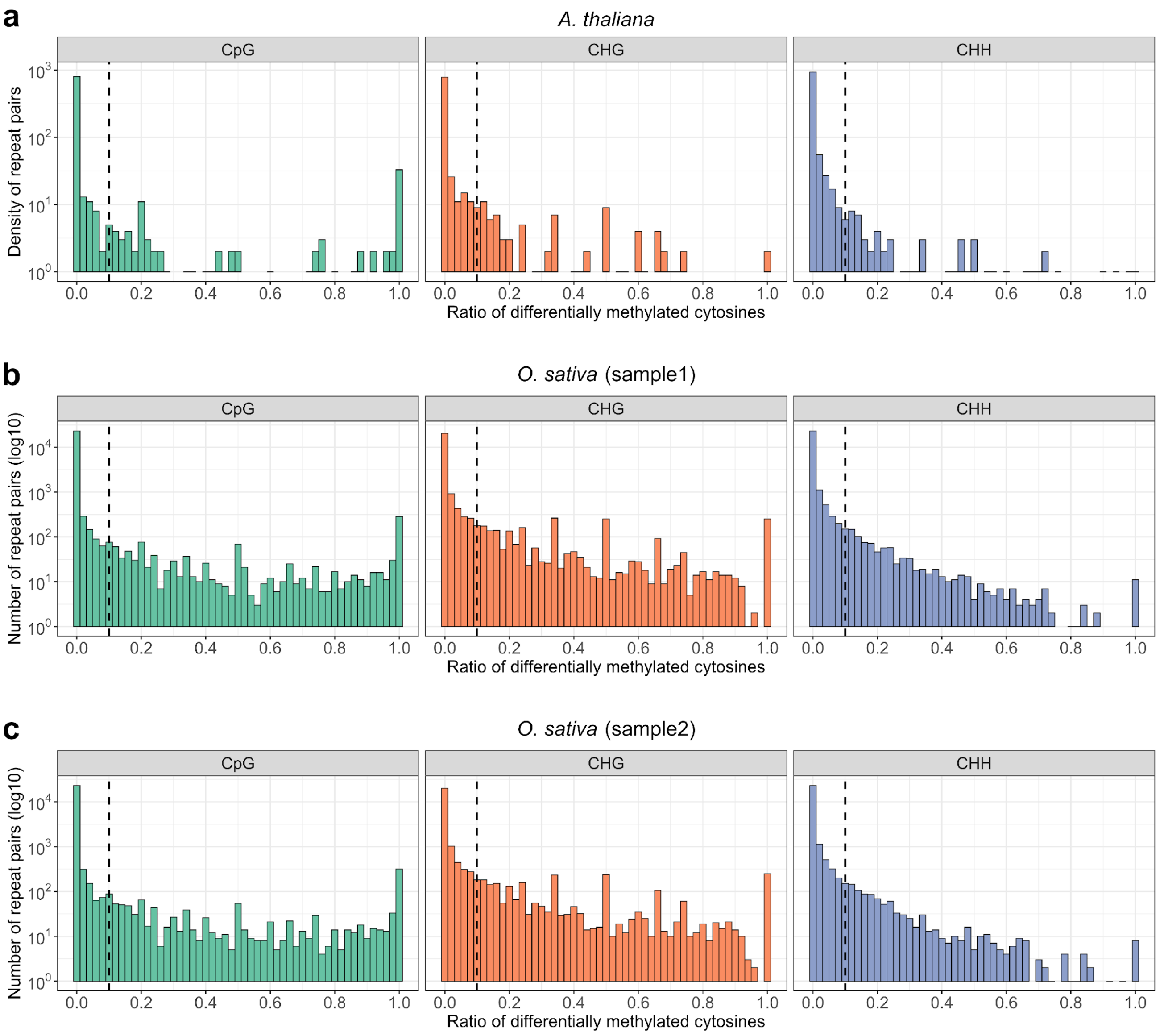


**Supplementary Fig. 25.** Ratio of differentially methylated CpG, CHG, and CHH sites to total CpG, CHG, and CHH sites respectively in each repeat pair of *A. thaliana* (**a**) and *O. sativa* sample1 (**b**) and *O. sativa* sample2 (**c**). The black dash lines (10%) indicate repeat pairs are differentially methylated (right) or not (left).

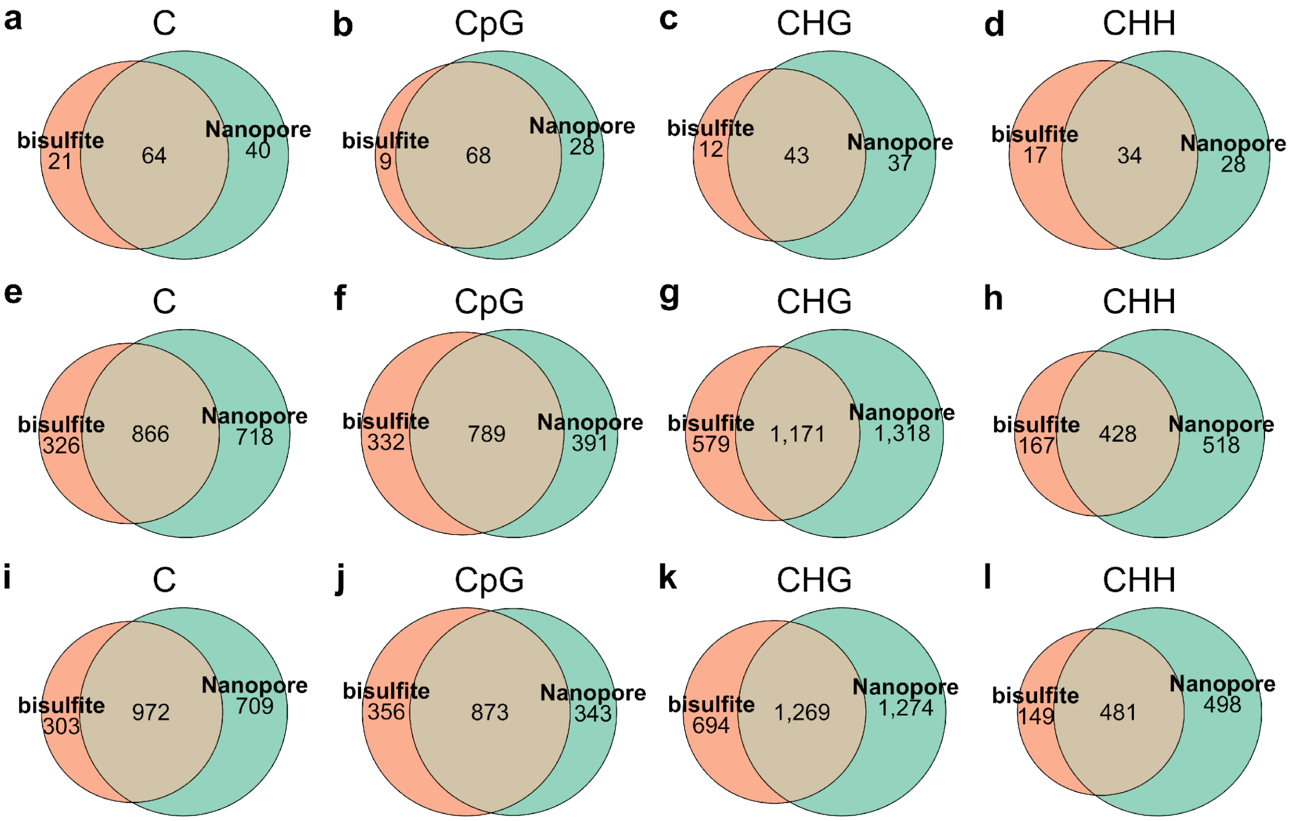


**Supplementary Fig. 26.** Comparison of differentially methylated repeat pairs profiled by bisulfite sequencing (Bismark) and Nanopore sequencing (DeepSignal-plant). **a-d:** Comparison of differentially methylated repeat pairs identified by methylation of cytosines (a), CpG sites (b), CHG sites (c), and CHH sites (d) in *A. thaliana*. **e-h:** Comparison of differentially methylated repeat pairs identified by methylation of cytosines (e), CpG sites (f), CHG sites (g), and CHH sites (h) in *O. sativa* (sample1). **i-l:** Comparison of differentially methylated repeat pairs identified by methylation of cytosines (i), CpG sites (j), CHG sites (k), and CHH sites (l) in *O. sativa* (sample2).

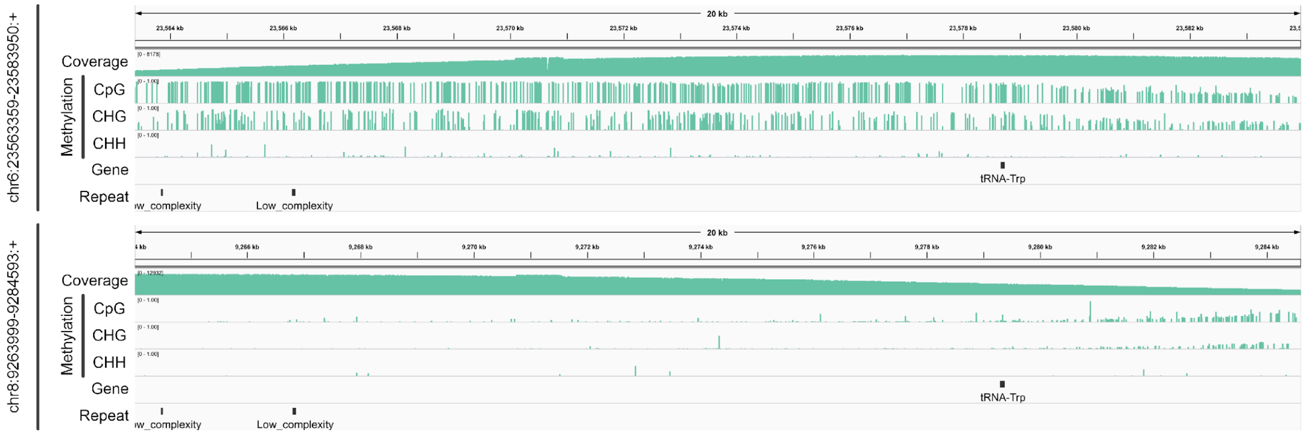


**Supplementary Fig. 27.** Genome browser view of a differentially methylated repeat pair (chr6:23563359-23583950:+, chr8: 9263999-9284593:+) in *O. sativa* (sample2). Source data are provided as a Source Data file.

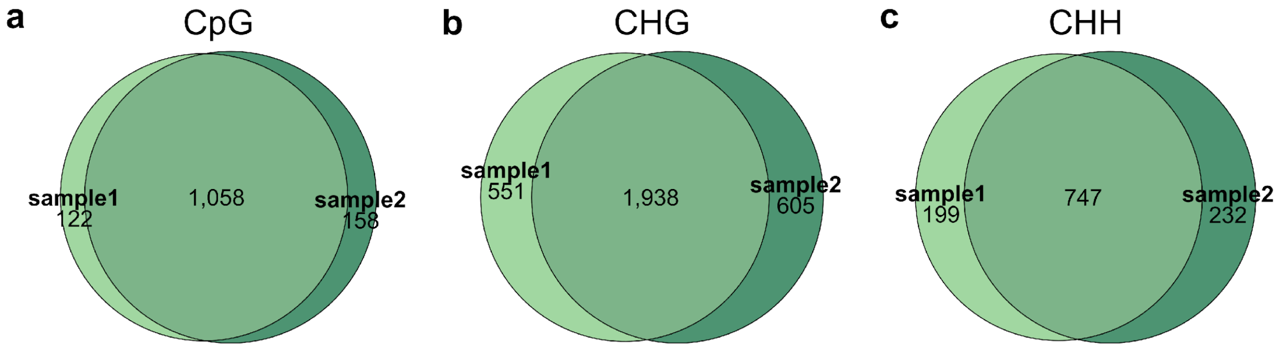


**Supplementary Fig. 28.** Comparison of differentially methylated repeat pairs in *O. sativa* sample1 and sample2 identified by methylation of CpG sites (**a**), CHG sites (**b**), and CHH sites (**c**) independently, which were detected by DeepSignal-plant.

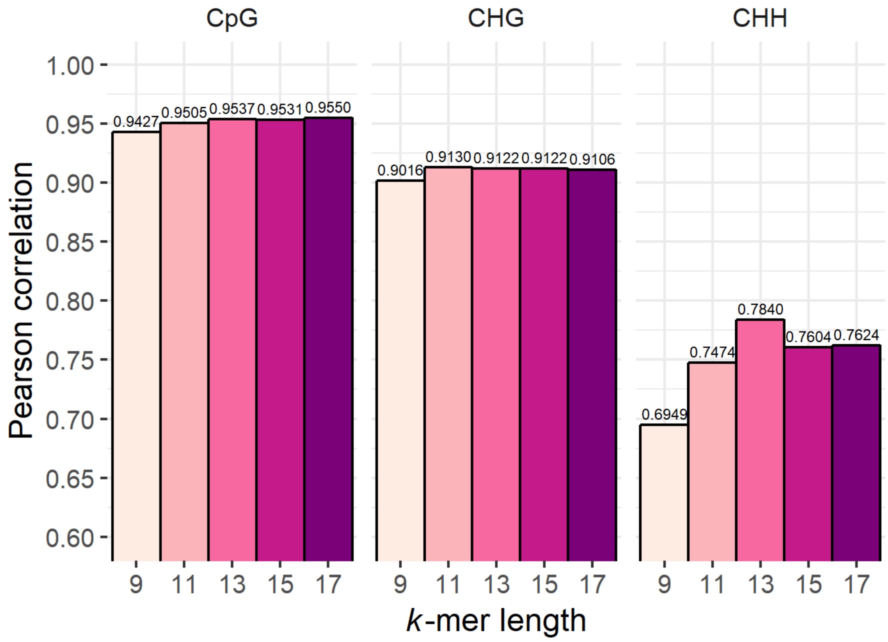


**Supplementary Fig. 29.** *k*-mer length tuning of DeepSignal-plant. The training samples are extracted from ~500× Nanopore reads of *A. thaliana*. Pearson correlations are calculated using the results from ~20× Nanopore reads and three bisulfite replicates of *A. thaliana*.

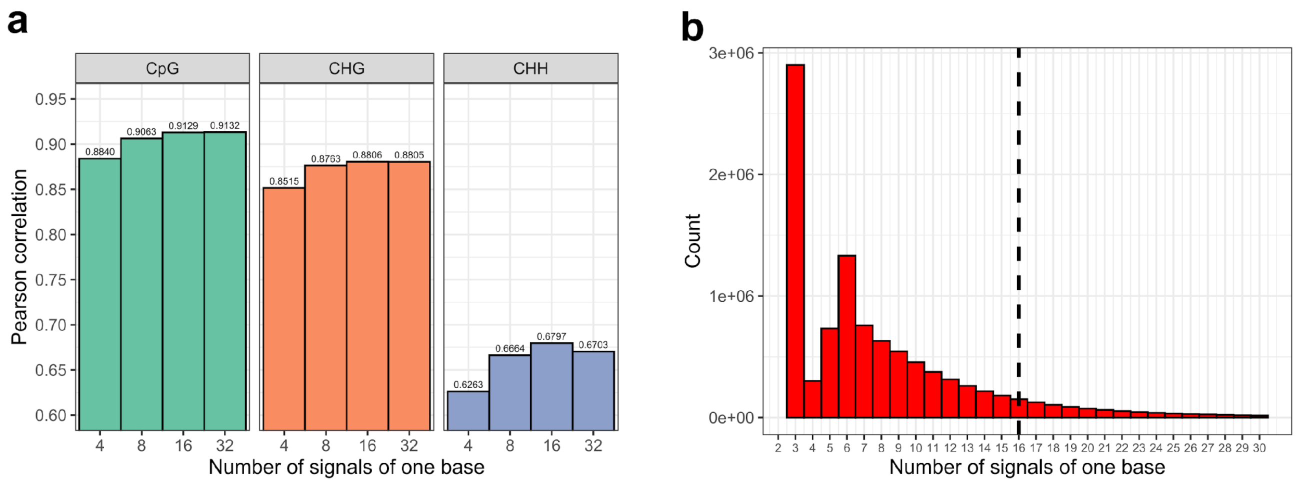


**Supplementary Fig. 30.** Selection of Number of signals of one base in DeepSignal-plant. **a:** Hyperparameter tuning on the number of signals of one base. Note that only signal features are used in DeepSignal-plant during the hyperparameter tuning. **b:** Number of signals of 10 million randomly selected bases. Suppose $u$ and $\sigma$ are mean and standard deviation of the number of signals, the dashed line indicates approximately $u+\sigma$ signals.

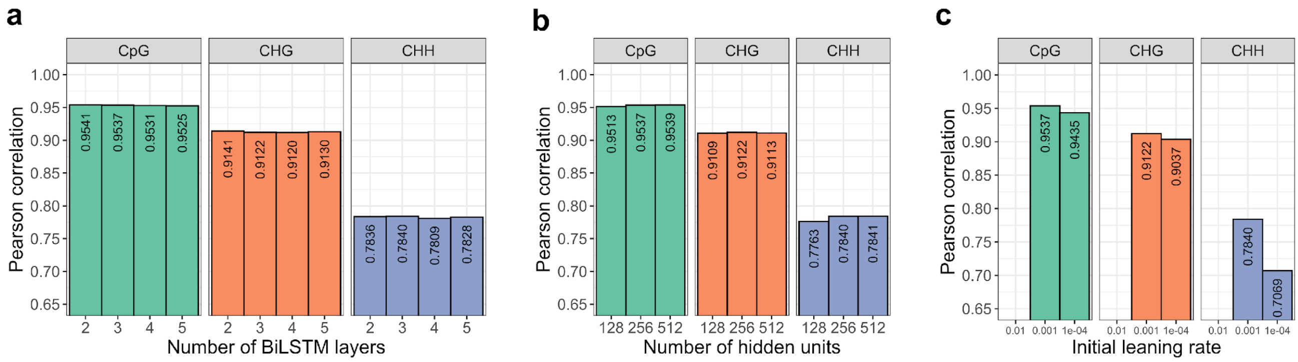


**Supplementary Fig. 31.** Hyperparameter tuning on the number of BiLSTM layers, the number of hidden units, and the initial learning rate of DeepSignal-plant.

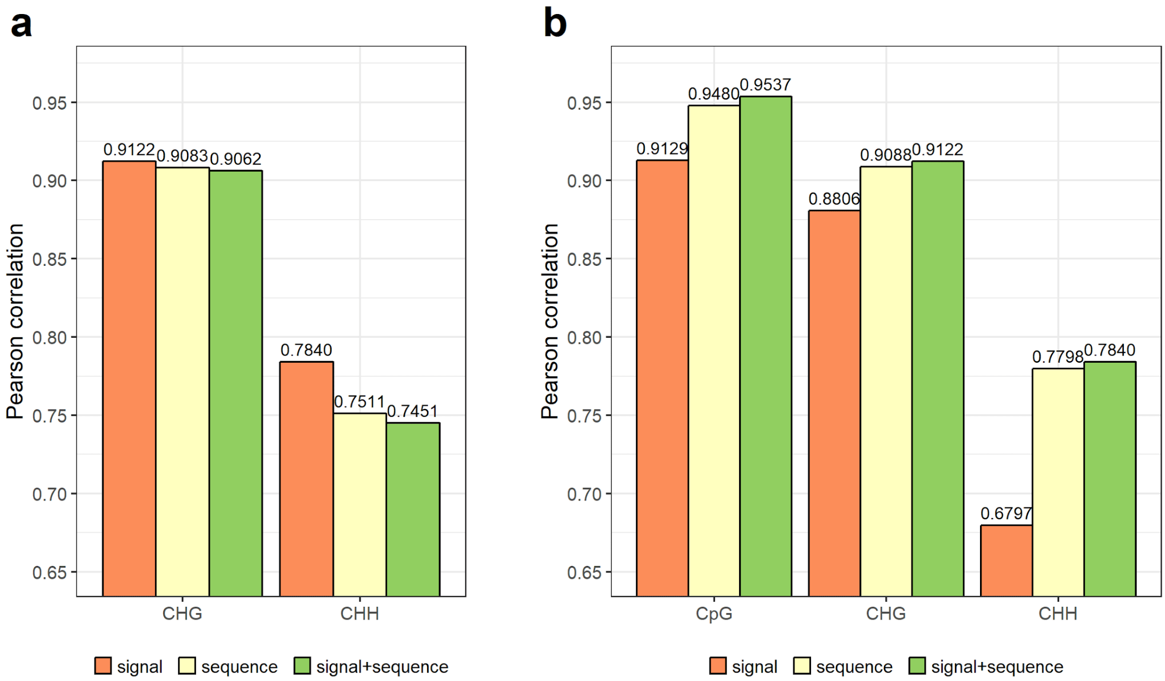


**Supplementary Fig. 32.** Feature selection of DeepSignal-plant to denoise training samples and call methylation (The training samples are extracted from ~500× Nanopore reads. Pearson correlations are calculated using the results from ~20× Nanopore reads and three bisulfite replicates of *A. thaliana*.). **a:** Evaluation of different features to denoise training samples (After denoising training samples, the training samples are used to training models for calling methylation using signal+sequence features). **b:** Evaluation of different features to call methylation (Before training, all the training samples are balanced first and then denoised (for CHG and CHH motif) using only signal features).

**Supplementary Table 1.** The number of CpG, CHG, CHH sites which bisulfite sequencing (Bismark) and Nanopore sequencing (DeepSignal-plant) can detect in *A. thaliana* and *O. sativa*. We count the sites from 5 chromosomes of *A. thaliana* genome and 12 chromosomes of *O. sativa* genome. Sites from both forward and complement strands of the genomes are counted. In bisulfite sequencing of *A. thaliana*, we count sites that satisfy *cov*>=1 or 5 in at least 1 replicate. In Nanopore sequencing, we count sites that satisfy *cov*>=1 or 5 from ~100x tested reads. *cov*=coverage.

| species | motif | genome | bisulfite | | Nanopore | |
|---|---|---|---|---|---|---|
| | | | *cov*>=1 | *cov*>=5 | *cov*>=1 | *cov*>=5 |
| *A. thaliana* | CpG | 5,567,714 | 5,487,342 | 5,468,996 | 5,549,652 | 5,521,044 |
| | CHG | 6,093,647 | 6,014,437 | 5,996,330 | 6,079,079 | 6,063,014 |
| | CHH | 31,198,155 | 30,774,058 | 30,653,400 | 31,106,922 | 31,011,252 |
| *O. sativa* (sample1) | CpG | 30,817,376 | 29,594,582 | 28,712,658 | 30,714,046 | 30,498,978 |
| | CHG | 27,376,461 | 26,418,299 | 25,767,463 | 27,316,646 | 27,196,935 |
| | CHH | 104,355,374 | 100,637,175 | 97,252,858 | 104,123,055 | 103,686,499 |
| *O. sativa* (sample2) | CpG | 30,817,376 | 29,755,811 | 28,542,701 | 30,711,556 | 30,486,515 |
| | CHG | 27,376,461 | 26,562,610 | 25,722,292 | 27,315,000 | 27,195,722 |
| | CHH | 104,355,374 | 101,378,360 | 98,481,274 | 104,117,684 | 103,686,107 |

**Supplementary Table 2.** The number of parameters in the model architecture of DeepSignal and DeepSignal-plant.

| feature source | DeepSignal | | DeepSignal-plant | |
|---|---|---|---|---|
| | architecture | No. of parameters | architecture | No. of parameters |
| sequence features | 3-layer BiLSTM | 3,026,944 | 1-layer BiLSTM + 1 fully connected layer | 173,248 |
| signal features | 11 inception blocks | 991,680 | 1-layer BiLSTM + 1 fully connected layer | 182,400 |
| concatenated | 2 fully connected layers | 36,397,088 | 3-layer BiLSTM + 2 fully connected layers | 4,338,434 |
| - | total | 40,415,712 | total | 4,694,082 |

**Supplementary Table 3.** The number of high-confidence sites in *A. thaliana* (3 technical replicates) and *O. sativa* (2 biological replicates). A site is considered to be methylated with high confidence if the site is covered with at least 5 reads and has at least 0.9 methylation frequency. A site is considered to be unmethylated with high confidence if it has at least five mapped reads and the methylation frequency is 0. Numbers in bold indicate the number of sites we select to train models.

| motif | state | *A. thaliana* | | | | | *O. sativa* | |
|---|---|---|---|---|---|---|---|---|
| | | replicate1 | replicate2 | replicate3 | intersection | union | sample1 | sample2 |
| CpG | methylated | 546,320 | 543,200 | 553,574 | **233,528** | 882,728 | **13,367,759** | 13,405,189 |
| | unmethylated | 3,120,040 | 3,019,453 | 3,111,175 | **2,257,533** | 3,630,296 | **9,380,609** | 10,053,549 |
| CHG | methylated | 35,512 | 34,258 | 36,253 | 12,482 | **65,076** | **2,845,705** | 2,833,756 |
| | unmethylated | 4,161,281 | 4,018,022 | 4,149,921 | **3,018,418** | 4,823,546 | **10,213,164** | 11,523,142 |
| CHH | methylated | 7,577 | 6,962 | 7,722 | 1,226 | **16,434** | **148,789** | 131,885 |
| | unmethylated | 22,285,718 | 21,400,918 | 22,212,460 | **15,717,929** | 26,382,803 | **53,916,702** | 63,447,979 |

**Supplementary Table 4.** Comparison of the number of unique *k*-mers in high-confidence methylated and unmethylated sites for training (*k*=13).

| motif | *A. thaliana* | | | *O. sativa* | | |
|---|---|---|---|---|---|---|
| | methylated | unmethylated | intersection | methylated | unmethylated | intersection |
| CpG | 179,687 | 1,343,098 | 88,496 | 2,635,394 | 2,808,801 | 1,905,205 |
| CHG | 43,713 | 1,386,150 | 29,856 | 736,876 | 2,503,090 | 637,048 |
| CHH | 13,107 | 5,294,855 | 10,354 | 65,033 | 8,340,086 | 62,066 |

**Supplementary Table 5.** Comparison of per-site methylation frequencies predicted by DeepSignal-plant and Megalodon from Nanopore sequencing with the results calculated from bisulfite sequencing in *A. thaliana* and *O. sativa*. ~100× Nanopore reads of *A. thaliana*, *O. sativa* (sample1), and *O. sativa* (sample2) were used, respectively. Models of DeepSignal-plant and Megalodon were trained using combined reads of *A. thaliana* and *O. sativa*. *r*: Pearson correlation; $r^2$: coefficient of determination; $\rho$: Spearman correlation; *RMSE*: root mean square error.

| species | motif | method | $r$ | $r^2$ | $\rho$ | *RMSE* |
|---|---|---|---|---|---|---|
| *A. thaliana* | CpG | DeepSignal-plant | 0.9850 | 0.9703 | 0.8253 | 0.0684 |
| | | Megalodon | 0.9597 | 0.9209 | 0.7948 | 0.1131 |
| | CHG | DeepSignal-plant | 0.9647 | 0.9306 | 0.7106 | 0.0567 |
| | | Megalodon | 0.9506 | 0.9036 | 0.7246 | 0.0675 |
| | CHH | DeepSignal-plant | 0.9045 | 0.8181 | 0.5795 | 0.0458 |
| | | Megalodon | 0.8126 | 0.6602 | 0.5501 | 0.0661 |
| *O. sativa* (sample1) | CpG | DeepSignal-plant | 0.9922 | 0.9844 | 0.8535 | 0.0618 |
| | | Megalodon | 0.9884 | 0.9768 | 0.8425 | 0.0708 |
| | CHG | DeepSignal-plant | 0.9666 | 0.9344 | 0.8615 | 0.0938 |
| | | Megalodon | 0.9677 | 0.9365 | 0.8975 | 0.0934 |
| | CHH | DeepSignal-plant | 0.8600 | 0.7396 | 0.5282 | 0.0643 |
| | | Megalodon | 0.8327 | 0.6934 | 0.4957 | 0.0672 |
| *O. sativa* (sample2) | CpG | DeepSignal-plant | 0.9921 | 0.9844 | 0.8636 | 0.0609 |
| | | Megalodon | 0.9852 | 0.9706 | 0.8514 | 0.0803 |
| | CHG | DeepSignal-plant | 0.9655 | 0.9321 | 0.8721 | 0.0957 |
| | | Megalodon | 0.9652 | 0.9315 | 0.9077 | 0.0957 |
| | CHH | DeepSignal-plant | 0.8718 | 0.7600 | 0.5575 | 0.0583 |
| | | Megalodon | 0.8362 | 0.6993 | 0.5016 | 0.0585 |

**Supplementary Table 6.** Comparison of per-site methylation frequencies predicted by DeepSignal-plant and Megalodon from Nanopore sequencing with the results calculated from bisulfite sequencing in *B. nigra*. ~78× Nanopore reads were used. Models of DeepSignal-plant and Megalodon were trained using combined reads of *A. thaliana* and *O. sativa*. *r*: Pearson correlation; $r^2$: coefficient of determination; $\rho$: Spearman correlation; *RMSE*: root mean square error.

| motif | method | $r$ | $r^2$ | $\rho$ | *RMSE* |
|---|---|---|---|---|---|
| CpG | DeepSignal-plant | 0.9702 | 0.9413 | 0.6878 | 0.1016 |
| | Megalodon | 0.9659 | 0.9329 | 0.6623 | 0.1035 |
| CHG | DeepSignal-plant | 0.8952 | 0.8015 | 0.8610 | 0.1355 |
| | Megalodon | 0.9010 | 0.8118 | 0.8717 | 0.1354 |
| CHH | DeepSignal-plant | 0.7536 | 0.5679 | 0.5547 | 0.1019 |
| | Megalodon | 0.7067 | 0.4994 | 0.5110 | 0.1158 |

**Supplementary Table 7.** Comparison of DeepSignal-plant and Megalodon at read level. Models of DeepSignal-plant and Megalodon were trained using combined reads of *A. thaliana* and *O. sativa*. AUC: Area Under the Curve. For each motif of each species, we randomly sampled 100,000 from each of the negative and positive datasets for evaluation and repeated 5 times. Values are averages of 5 replicated tests.

| species | motif | method | accuracy | sensitivity | specificity | AUC |
|---|---|---|---|---|---|---|
| *A. thaliana* | CpG | DeepSignal-plant | 0.9266 | 0.8873 | 0.9659 | 0.9702 |
| | | Megalodon | 0.8744 | 0.7805 | 0.9682 | 0.9506 |
| | CHG | DeepSignal-plant | 0.9327 | 0.8688 | 0.9890 | 0.9687 |
| | | Megalodon | 0.8870 | 0.7770 | 0.9913 | 0.9670 |
| | CHH | DeepSignal-plant | 0.8696 | 0.7472 | 0.9920 | 0.9525 |
| | | Megalodon | 0.7560 | 0.5163 | 0.9958 | 0.9196 |
| *O. sativa* (sample1) | CpG | DeepSignal-plant | 0.9556 | 0.9472 | 0.9640 | 0.9900 |
| | | Megalodon | 0.9543 | 0.9262 | 0.9823 | 0.9879 |
| | CHG | DeepSignal-plant | 0.9501 | 0.9125 | 0.9878 | 0.9812 |
| | | Megalodon | 0.9302 | 0.8710 | 0.9894 | 0.9783 |
| | CHH | DeepSignal-plant | 0.9287 | 0.8698 | 0.9876 | 0.9723 |
| | | Megalodon | 0.8545 | 0.7222 | 0.9867 | 0.9669 |
| *O. sativa* (sample2) | CpG | DeepSignal-plant | 0.9533 | 0.9443 | 0.9624 | 0.9890 |
| | | Megalodon | 0.9495 | 0.9192 | 0.9798 | 0.9861 |
| | CHG | DeepSignal-plant | 0.9507 | 0.9152 | 0.9863 | 0.9816 |
| | | Megalodon | 0.9288 | 0.8706 | 0.9870 | 0.9780 |
| | CHH | DeepSignal-plant | 0.9296 | 0.8725 | 0.9867 | 0.9777 |
| | | Megalodon | 0.8479 | 0.7130 | 0.9828 | 0.9689 |
| *B. nigra* | CpG | DeepSignal-plant | 0.9257 | 0.9316 | 0.9199 | 0.9784 |
| | | Megalodon | 0.9394 | 0.9114 | 0.9674 | 0.9806 |
| | CHG | DeepSignal-plant | 0.9030 | 0.8443 | 0.9617 | 0.9455 |
| | | Megalodon | 0.8856 | 0.7972 | 0.9741 | 0.9500 |
| | CHH | DeepSignal-plant | 0.6938 | 0.4135 | 0.9742 | 0.7679 |
| | | Megalodon | 0.6420 | 0.2990 | 0.9850 | 0.7430 |

**Supplementary Table 8.** Comparison of *k*-mers in the training dataset of DeepSignal-plant and in the regions which can only be covered by Nanopore sequencing (*k*: 13; NO.: number of *k*-mers; overlap ratio: ratio of *k*-mers in corresponding regions which are also in training dataset).

| motif | training dataset | regions that can only be covered by Nanopore sequencing | | | | | |
|---|---|---|---|---|---|---|---|
| | | *A. thaliana* | | *O. sativa* (sample1) | | *O. sativa* (sample2) | |
| | No. | No. | overlap ratio | No. | overlap ratio | No. | overlap ratio |
| CpG | 3,554,085 | 42,742 | 91.5% | 359,862 | 96.0% | 372,326 | 97.0% |
| CHG | 2,645,372 | 44,315 | 91.9% | 317,515 | 94.8% | 301,492 | 94.8% |
| CHH | 5,934,274 | 215,100 | 80.3% | 1,474,849 | 85.1% | 1,085,718 | 83.2% |

**Supplementary Table 9.** The number of repeat pairs in *A. thaliana* and *O. sativa*. Differentially methylated repeat pairs are based on the results of DeepSignal-plant. “total” counts repeat pairs which contain at least 1 cytosine in the corresponding motif. “differential” counts repeat pairs which contain at least 10% differentially methylated cytosines in the corresponding motif.

| species | motif | repeat pairs | | | | | |
|---|---|---|---|---|---|---|---|
| | | length>=100 | | length>=1000 | | length>=10000 | |
| | | total | differential | total | differential | total | differential |
| *A. thaliana* | C | 1,104 | 104 | 356 | 9 | 46 | 0 |
| | CpG | 936 | 96 | 356 | 28 | 46 | 0 |
| | CHG | 938 | 80 | 356 | 13 | 46 | 0 |
| | CHH | 1,103 | 62 | 356 | 3 | 46 | 0 |
| *O. sativa* | C | 26,508 | 1,584 | 10,358 | 239 | 256 | 6 |
| (sample1) | CpG | 24,964 | 1,180 | 10,358 | 356 | 256 | 19 |
| | CHG | 24,941 | 2,489 | 10,358 | 461 | 256 | 10 |
| | CHH | 26,476 | 946 | 10,358 | 42 | 256 | 0 |
| *O. sativa* | C | 26,508 | 1,681 | 10,358 | 250 | 256 | 5 |
| (sample2) | CpG | 24,964 | 1,216 | 10,358 | 355 | 256 | 16 |
| | CHG | 24,941 | 2,543 | 10,358 | 477 | 256 | 9 |
| | CHH | 26,476 | 979 | 10,358 | 34 | 256 | 0 |

**Supplementary Table 10.** The number of reads in *A. thaliana*, *O. sativa*, and *B.nigra* used for training and testing.

| species | No. reads | |
|---|---|---|
| | training | testing |
| *A. thaliana* | 2,587,533 | 537,075 |
| *O. sativa* (sample1) | 1,696,000 | 1,578,036 |
| *O. sativa* (sample2) | - | 1,671,237 |
| *B. nigra* | - | 6,317,961 |

**Supplementary Table 11.** Default hyperparameters of DeepSignal-plant. Note that number of layers indicates the number of BiLSTM layers to process concatenated (sequence + signal) features.

| parameter | value |
|---|---|
| length of *k*-mer | 13 |
| *m* (number of signals) | 16 |
| number of layers | 3 |
| number of hidden units | 256 |
| initial learning rate | 0.001 |

**Supplementary Table 12.** Running time and peak memory usage of the pipeline of DeepSignal-plant on Nanopore data. Note that time means real wall-clock time; memory means peak memory.

| species | No. reads | basecall (Guppy) | | re-squiggle (Tombo) | | call methylation (DeepSignal-plant) | |
|---|---|---|---|---|---|---|---|
| | | time (h:m:s) | memory (GB) | time (h:m:s) | memory (GB) | time (h:m:s) | memory (GB) |
| *A. thaliana* | 537,075 | 10:55:49 | 7.2 | 11:47:4 | 12.5 | 38:56:24 | 53.3 |
| *O. sativa* (sample1) | 1,578,036 | 42:57:35 | 7.1 | 71:4:2 | 47.4 | 185:56:4 | 67.4 |
| *O. sativa* (sample2) | 1,671,237 | 44:4:56 | 7.3 | 74:16:37 | 47.5 | 185:2:10 | 67.4 |
| *B. nigra* | 6,317,961 | 51:43:37 | 6.7 | 84:54:46 | 58.3 | 156:36:33 | 73.2 |

## Supplementary Note 1. Simulation experiment to evaluate the denoising method

To validate the denoising method, we performed a simulation experiment using our *A. thaliana* sequencing data as follows:

(1) We first establish ground-truth datasets. Based on bisulfite sequencing, we select cytosines with methylation frequencies equal to 1 and 0. Then for each motif, we extracted the corresponding true-positive and true-negative samples of the selected sites from Nanopore reads. We generate 9,388,125, 972,099, and 309,301 true-positive samples for CpG, CHG, and CHH, respectively. To establish a ground-truth dataset for each motif, we use the balancing method to generate balanced positive and negative training samples.

(2) For each motif, we randomly change the labels of the certain number of negative samples from 0 (negative) to 1 (positive) in the ground-truth dataset and remove the same number of true-positive samples, to generate datasets with different mislabeled ratios (0%, 5%, 10%, 15%, and 20%). For example, in a dataset with a 10% mislabeled ratio, 10% of positive samples are mislabeled samples (i.e., false-positive samples), while the total number of positive samples are still 9,388,125, 972,099, and 309,301 for CpG, CHG, and CHH, respectively. Then, we evaluate the denoising method using the datasets. We repeat 5 times the mislabeling-denoising experiment for each mislabel ratio.

The results show that, although a small portion of true-positive samples is removed, most of the mislabeled samples are removed by the denoising method. For example, in the datasets with a 10% mislabeled ratio, 15.3% (CG), 17.8% (CHG), 35.4% (CHH) true-positive samples are removed, while 96.9% (CG), 97.5% (CHG), 94.8% (CHH) mislabeled samples are removed.

## Supplementary Note 2. The model architecture of DeepSignal-plant

(1) A bidirectional LSTM layer

A bidirectional LSTM layer includes a forward LSTM and a backward LSTM to catch both forward and reverse flow of features. Let $x_1$, $x_2$,..., $x_t$ are a sequence of features. For sequence features used in DeepSignal-plant, each time step $x_i$ contains four features: the nucleotide base, the mean, standard deviation and the number of mapped signals of the base. For signal features in DeepSignal-plant, each time step $x_i$ contains *m* features which are *m* signals of the current base. A LSTM will recursively calculate the hidden layer *h* as follows:

$$i_t = sigmoid(W_{xi}x_t + W_{hi}h_{t-1} + W_{ci}\odot c_{t-1} + b_i) \quad \textbf{(1)}$$

$$f_t = sigmoid(W_{xf}x_t + W_{hf}h_{t-1} + W_{cf}\odot c_{t-1} + b_f) \quad \textbf{(2)}$$

$$c_t = f_t \odot c_{t-1} + i_t \odot tanh(W_{xc}x_t + W_{hc}\odot c_t + b_c) \quad \textbf{(3)}$$

$$o_t = sigmoid(W_{xo}x_t + W_{ho}h_{t-1} + W_{co}\odot c_t + b_o) \quad \textbf{(4)}$$

$$h_t = o_t \odot tanh(c_t) \quad \textbf{(5)}$$

where *W* and *b* are weights and biases in the model. *x* is the input vector; *i* is the activation vector of the input gate; *f* is the activation vector of the forget gate; *c* is the cell state vector; *o* is the activation vector of the output gate; and *h* is the output vector of the LSTM hidden unit. Current output $h_t$ of LSTM hidden unit depends on the input $x_t$, the previous state $h_{t-1}$, and previous information stored in a cell. Then, the outputs of forward and backward LSTM are combined:

$$z_t = h_{t,F} \oplus h_{t,B} \quad \textbf{(6)}$$

(2) Softmax activation function

In DeepSignal-plant, softmax activation function is used to predict the methylated and unmethylated probabilities of one sample as follows:

$$softmax(x_i) = \frac{e^{x_i}}{\sum_{j=0}^{1} e^{x_j}},\ i = 0\ or\ 1 \quad \textbf{(7)}$$

where $x_0$ and $x_1$ are two outputs from the former fully connected layer.

(3) The cross-entropy loss function used during training is as follows:

$$L = z * - log(y) + (1 - z) * - log(1 - y) \quad \textbf{(8)}$$

where $z$ is the true label vector and $y$ is the predicted probability vector output from the softmax function.

## Supplementary Note 3. Hyperparameters and feature selection of DeepSignal-plant

DeepSignal-plant applies bidirectional long short-term memory (BiLSTM) layers to detect methylation. We use one BiLSTM layer to receive sequence features and signal features, respectively. Three BiLSTM layers are used to process the concatenated features. Using *A. thaliana* data ( ~500× reads for training and another ~20×reads for testing), we tune the hyperparameters of DeepSignal-plant: the length of $k$-mer, the number of signals $m$, the number of BiLSTM layers to process the concatenated sequence and signal features, the number of hidden units in each BiLSTM layer, and the initial learning rate for training. We use control variables to test the effect of each hyperparameter. *i.e.*, to test different values of a single hyperparameter, we set other hyperparameters as the default values (Supplementary Table 11). According to the results, we set the length of $k$-mer $k$=13 (Supplementary Fig. 29), the number of signals m=16 (Supplementary Fig. 30a). By testing the number of signals of 10 million bases randomly selected from reads of *A. thaliana*, we find that the number of signals of 91.4% bases is less than 16 (Supplementary Fig. 30b). The results of hyperparameter tuning on the number of BiLSTM layers, the number of hidden units, and the initial learning rate are shown in Supplementary Fig. 31. Note that the initial learning rate of 0.01 does not make the loss converge in training.

During training, we use a dropout probability of 0.5 at each dropout layer. We use a batch size of 512 and an initial learning rate of 0.001. The learning rate is adopted by the Adam optimizer and decayed by a factor of 0.1 after every two epochs. The parameter *betas* in Adam optimizer are set to (0.9, 0.999) as default. We train at least 5 epochs and at most 10 epochs during each training process.

DeepSignal-plant uses two groups of features (sequence features and signal features) to predict the methylation state of the one targeted site. We further use the reads of *A. thaliana* to test the effectiveness of the two groups of features in denoising training samples and calling methylation. As shown in Supplementary Fig. 32a, for CHG and CHH motif, using signal features to denoise training samples gets the best performances. For calling methylation of all three motifs, using signal features gets the worst performance, and using both features gets the highest performance (Supplementary Fig. 32b).

## Supplementary Note 4. Running time and memory usage of the DeepSignal-plant pipeline

We evaluate the running time and peak memory of three main steps in the pipeline of DeepSignal-plant: (1) Basecall using Guppy; (2) Re-squiggle using Tombo; (3) Call methylation using DeepSignal-plant. The data used for evaluation include 100× (mean genome coverage) *A. thaliana* Nanopore reads, 100× *O. sativa* (sample1) Nanopore reads, 100× *O. sativa* (sample2) Nanopore reads, and 78× *B. nigra* Nanopore reads. We process all data at a server with 40 CPU processors (Intel(R) Xeon(R) CPU E5-2676 v3 @ 2.40GHz), 256 GB RAM, and a 12GB TITAN X (Pascal) GPU. For basecalling using Guppy, we use 1 cpu processor and 1 GPU. For re-squiggling using Tombo, we use 40 cpu processors. For methylation calling using DeepSignal-plant, we use 40 cpu processors and 1 GPU. The running time and peak memory of these three steps were shown in Supplementary Table 12.

## Supplementary Note 5. Model training and modified base calling in Megalodon

Megaldon uses models of Guppy for modified base calling. During the modified base calling, Megalodon treats a modified base as a new base (Z by default), which is different from the regular ACGT(U) bases. To train a new model, an initial model needs to be provided (Supplementary Fig. 4b) [1]: (1) Modified base calling by the initial model. To prepare training data from a set of Nanopore reads for a new model, the Nanopore reads must be called by the initial model first. (2) Ground truth aided bootstrap modified base annotation. Given the results called by the initial model and the methylation profile from bisulfite sequencing, Megalodon generates a modified base threshold for each targeted base. (3) Generating of signals-based mapping. Using the modified base annotation, a second time run of modified base calling is performed to get the mapping between the raw signals and the reference sequences for each read. (4) Model training. Using the signals-based mapping data, Taiyaki [2] is used to train a model of Guppy. The new model can be used for

modified base calling by both Guppy and Megalodon.

Modified base calling of Megalodon contains three main steps: (1) Basecalling. Megalodon uses Guppy to basecall. During the basecalling of Guppy, the raw reads are processed with a recurrent neural network, and then are decoded with Viterbi decoding. Megalodon gets nucleotide sequences, and the link between the called bases and the neural network outputs from Guppy. (2) Reference anchoring. After basecalling, Megalodon uses minimap2 to align reads to the reference sequence[3]. Thus, the neural network outputs are also anchored to the reference sequence. (3) Modified base calling. For each targeted base, Megalodon extracts a local context sequence around the targeted base. Then Megalodon performs a scoring algorithm (forward-backward algorithm and Viterbi decoding) over the corresponding local neural network output, to find the best path (path with or without a modified base) for classification.

## Supplementary references

[1]Oxford Nanopore Technologies. Megalodon. https://nanoporetech.github.io/megalodon/modbase_training.html. Accessed 25 June 2021.
[2]Oxford Nanopore Technologies. Taiyaki. https://github.com/nanoporetech/taiyaki. Accessed 25 June 2021.
[3]Li, H. Minimap2: pairwise alignment for nucleotide sequences. *Bioinformatics* **34**, 3094-3100 (2018).
