# Supplementary material for: Genome-wide detection of cytosine methylations in plant from Nanopore data using deep learning
Source: Nat Commun. 2021 Oct 13;12:5976. doi: 10.1038/s41467-021-26278-9 (PMC8514461; doi:10.1038/s41467-021-26278-9)
Supplement: Supplementary file 7 — Reporting summary [file 41467_2021_26278_MOESM7_ESM.pdf]

nature portfolio

Corresponding author(s): Chuan-Le Xiao, Feng Luo, Jianxin Wang

Last updated by author(s): Sep 21, 2021

Reporting Summary

Nature Portfolio wishes to improve the reproducibility of the work that we publish. This form provides structure for consistency and transparency in reporting. For further information on Nature Portfolio policies, see our Editorial Policies and the Editorial Policy Checklist.

Statistics

For all statistical analyses, confirm that the following items are present in the figure legend, table legend, main text, or Methods section.

n/a	Confirmed	
☐	☒	The exact sample size (*n*) for each experimental group/condition, given as a discrete number and unit of measurement
☐	☒	A statement on whether measurements were taken from distinct samples or whether the same sample was measured repeatedly
☒	☐	The statistical test(s) used AND whether they are one- or two-sided<br>*Only common tests should be described solely by name; describe more complex techniques in the Methods section.*
☒	☐	A description of all covariates tested
☒	☐	A description of any assumptions or corrections, such as tests of normality and adjustment for multiple comparisons
☐	☒	A full description of the statistical parameters including central tendency (e.g. means) or other basic estimates (e.g. regression coefficient) AND variation (e.g. standard deviation) or associated estimates of uncertainty (e.g. confidence intervals)
☒	☐	For null hypothesis testing, the test statistic (e.g. *F*, *t*, *r*) with confidence intervals, effect sizes, degrees of freedom and *P* value noted<br>*Give P values as exact values whenever suitable.*
☒	☐	For Bayesian analysis, information on the choice of priors and Markov chain Monte Carlo settings
☒	☐	For hierarchical and complex designs, identification of the appropriate level for tests and full reporting of outcomes
☐	☒	Estimates of effect sizes (e.g. Cohen's *d*, Pearson's *r*), indicating how they were calculated

Our web collection on statistics for biologists contains articles on many of the points above.

Software and code

Policy information about availability of computer code

Data collection	Nanopore data were sequenced on a PromethION sequencer (ONT) and collected from MinKNOW software (v19.06.8, ONT). The raw electrical data in fast5 format were converted to fastq format using Guppy basecaller (version 3.6.1+249406c) .
Data analysis	This manuscripts utilized open software and our shell scripts described in the Methods section and supplementary note. DeepSignal-plant (v0.1.2), Tandem Repeats Finder (version 4.09) and Inverted Repeats Finder (version 3.05), Guppy (version 3.6.1+249406c), Tombo (version 1.5.1) , minimap2 (version 2.17-r941) , Python3, PyTorch (version 1.2.0), scikit-learn (version 0.20.1), Megalodon (version 2.2.3), MUMmer (version 4.0.0beta2), Bismark (v0.20.0).

For manuscripts utilizing custom algorithms or software that are central to the research but not yet described in published literature, software must be made available to editors and reviewers. We strongly encourage code deposition in a community repository (e.g. GitHub). See the Nature Portfolio guidelines for submitting code & software for further information.

Data

Policy information about availability of data

All manuscripts must include a data availability statement. This statement should provide the following information, where applicable:

- Accession codes, unique identifiers, or web links for publicly available datasets
- A description of any restrictions on data availability
- For clinical datasets or third party data, please ensure that the statement adheres to our policy

All sequencing data generated in this study (bisulfite sequencing and Nanopore sequencing data of A. thaliana and O. sativa) have been deposited in the National Center for Biotechnology Information (NCBI) under BioProjectID PRJNA764549 (https://www.ncbi.nlm.nih.gov/bioproject/?term=PRJNA764549) and Sequence Read Archive (SRA) accession No. SRP337810 (https://trace.ncbi.nlm.nih.gov/Traces/sra/?study=SRP337810), as well as in the Genome Sequence Archive of BIG Data

Center, Beijing Institute of Genomics (BIG, http://gsa.big.ac.cn), Chinese Academy of Sciences, with Project accession No. PRJCA004326 (https://ngdc.cncb.ac.cn/bioproject/browse/PRJCA004326) and GSA accession No. CRA003885 (https://ngdc.cncb.ac.cn/gsa/browse/CRA003885). Nanopore and bisulfite sequencing data of B. nigra are available at NCBI BioProject ID PRJNA516907 (https://www.ncbi.nlm.nih.gov/bioproject/?term=PRJNA516907). The gene annotation of A. thaliana is available at https://www.arabidopsis.org/index.jsp. The gene annotation of O. sativa is available at https://plants.ensembl.org/Oryza_sativa/Info/Index and https://rapdb.dna.affrc.go.jp/index.html.

Field-specific reporting

Please select the one below that is the best fit for your research. If you are not sure, read the appropriate sections before making your selection.

☒ Life sciences ☐ Behavioural & social sciences ☐ Ecological, evolutionary & environmental sciences

For a reference copy of the document with all sections, see nature.com/documents/nr-reporting-summary-flat.pdf

Life sciences study design

All studies must disclose on these points even when the disclosure is negative.

Sample size	Sample sizes were not predetermined but to train and evaluate our proposed method and other tools, for A. thaliana, we have sequenced three technical replicates using bisulfite sequencing. For O. sativa, we have sequenced two biological replicates using bisulfite sequencing. We performed one Nanopore sequencing of the same A. thaliana sample used in bisulfite sequencing and performed Nanopore sequencing of the two biological O. sativa samples.
Data exclusions	"Fail" Nanopore sequencing reads (mean Q-score <= 9) of A. thaliana and O. sativa were not used in the analysis.
Replication	I have three technical replicates of A. thaliana and two biological replicates of O. sativa.
Randomization	Samples were not randomized but each sample were sequenced in parallel using bisulfite and Nanopore sequencing, to evaluate different tested tools.
Blinding	Blinding was not relevant to this study, as different tested tools was evaluated by comparing against each other and the ground truth.

Reporting for specific materials, systems and methods

We require information from authors about some types of materials, experimental systems and methods used in many studies. Here, indicate whether each material, system or method listed is relevant to your study. If you are not sure if a list item applies to your research, read the appropriate section before selecting a response.

Materials & experimental systems

n/a	Involved in the study
☒	☐ Antibodies
☒	☐ Eukaryotic cell lines
☒	☐ Palaeontology and archaeology
☒	☐ Animals and other organisms
☒	☐ Human research participants
☒	☐ Clinical data
☒	☐ Dual use research of concern

Methods

n/a	Involved in the study
☒	☐ ChIP-seq
☒	☐ Flow cytometry
☒	☐ MRI-based neuroimaging
